# Ecdysone Mediates the Development of Immunity in the *Drosophila* Embryo

**DOI:** 10.1016/j.cub.2014.03.062

**Published:** 2014-05-19

**Authors:** Kiri Louise Tan, Isabella Vlisidou, Will Wood

**Affiliations:** 1 Faculty of Medical and Veterinary Sciences, University of Bristol, University Walk, Bristol BS8 1TD, UK

## Abstract

Beyond their role in cell metabolism, development, and reproduction, hormones are also important modulators of the immune system. In the context of inflammatory disorders, systemic administration of pharmacological doses of synthetic glucocorticoids (GCs) is widely used as an anti-inflammatory treatment [1, 2]. However, not all actions of GCs are immunosuppressive, and many studies have suggested that physiological concentrations of GCs can have immunoenhancing effects [3–7]. For a more comprehensive understanding of how steroid hormones regulate immunity and inflammation, a simple in vivo system is required. The *Drosophila* embryo has recently emerged as a powerful model system to study the recruitment of immune cells to sterile wounds [8] and host-pathogen dynamics [9]. Here we investigate the immune response of the fly embryo to bacterial infections and find that the steroid hormone 20-hydroxyecdysone (20-HE) can regulate the quality of the immune response and influence the resolution of infection in *Drosophila* embryos.

## Highlights

• *Drosophila* embryos respond to different infections via Imd and Toll signaling
• The immune response in the embryo requires the steroid hormone ecdysone
• The trachea is the primary immunoresponsive tissue in the embryonic humoral response

## Teaser

Tan et al. report that the immune response of *Drosophila* embryos is regulated by the steroid hormone ecdysone. Stage 15 embryos are able to respond to different types of infection via the Imd and Toll signaling pathways, rapidly switching on antimicrobial peptide production in the developing tracheal epithelium to overcome infection.

## Results and Discussion

### *Drosophila* Embryos Can Mount an Immune Response to Bacterial Challenge

Using a previously established embryo microinjection assay [9], we first sought to determine whether late-stage *Drosophila* embryos are able to induce a humoral immune response after septic injury. The humoral response to microbe infection in *Drosophila* is largely mediated by two pathways: Imd and Toll. Pathogen recognition is initiated by pattern recognition receptors that bind conserved stereotypical, rather than particular, molecular structures present in a wide spectrum of microorganisms but absent in the host [10], such as peptidoglycan (PGN), which is a major constituent of the cell wall of both Gram-negative and Gram-positive bacteria [11]. *Drosophila* senses mesodiaminopimelic acid (DAP)-type PGN present in a single layer within the Gram-negative bacteria periplasmic space via two peptidoglycan recognition proteins (PGRPs), the membrane-bound PGRP-LC and the secreted and/or cytosolic PGRP-LE, activating the Imd signaling pathway. Sensing of the Lys-type peptidoglycan present on the surface of Gram-positive bacteria is mediated by PGRP-SA, PGRP-SD, and Gram-negative binding protein 1 (GNBP1), which relay the signal to the Toll pathway [12]. A subclass of Gram-positive bacteria including *Bacillus* species and *Listeria monocytogenes* also produce DAP-type PGN, which renders them able to activate the Imd signaling pathway [12]. Activation of antimicrobial peptide (AMP) genes and quantification of pathogen load are widely used in both larval and adult fly models as readouts of the immune response. As a proxy for AMP production, we monitored the expression of a *Drosocin*-*gfp* (*Drc*-GFP) promoter fusion construct in stage 15 embryos injected with either *Escherichia coli* (*E. coli*) or *Erwinia carotovora carotovora 15* (*Ecc15*) [13].

**Figure 1:**
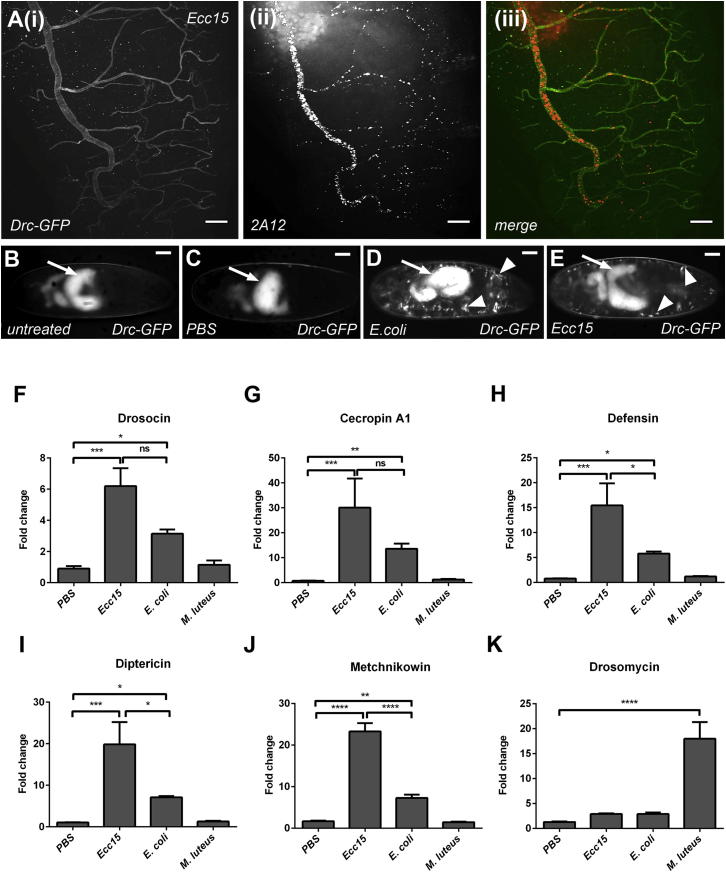
Stage 15 *Drosophila* Embryos Are Able to Mount Immune Responses to Bacterial Challenge (A–E) A stage 15 embryo expressing Drosocin-GFP stained with anti-GFP (Ai) and the tracheal-specific antibody 2A12 (Aii). The merge (Aiii) shows clear Drc expression throughout the tracheal network 3 hpi with *Ecc15*. A live, untreated, Drc-GFP-expressing embryo (B) and a Drc-GFP-expressing embryo (C) 6 hpi with PBS show no Drc expression, whereas injection with either *E. coli* (D) or *Ecc15* (E) leads to robust expression in the embryonic epithelium (arrowheads). Arrows show autofluorescence in the yolk. Scale bars represent 20 μm (A) and 50 μm (B–E). (F–K) Real-time qPCR analysis of *Drosocin* (F), *Cecropin A1* (G), *Defensin* (H), *Diptericin* (I), *Metchnikowin* (J), and *Drosomycin* (K) in stage 15 embryos injected with endotoxin-free PBS or live bacterial cells of *E. coli*, *M. luteus*, and *E. carotovora* (*Ecc15*) for 2 hr. The expression of antimicrobial peptide genes was normalized to the reference gene *rp49* and then standardized to the expression level of nontreated samples. The mean of three independent biological replicates is shown, and error bars represent the SD. ^∗^p < 0.05, ^∗∗^p < 0.01, and p^∗∗∗^ < 0.001 as determined by one-way ANOVA with an ad hoc Tukey’s multiple comparison test. n = 200 embryos.

Infection with *E. coli* or *Ecc15* initially induces *Drc-*GFP transgene expression throughout the tracheal system as early as 3 hr postinfection (hpi) (Figure 1A). This is followed by a later expression in the epithelium at 6 hpi (Figures 1D and 1E). The microinjection process itself had no effect on Drosocin expression (Figure 1C). To assess the early effects of septic injury on stage 15 embryos in more detail, we analyzed the transcriptional response of several AMP genes, including *Cecropin A1*, *Defensin*, *Diptericin*, *Drosocin*, *Drosomycin*, and *Metchnikowin*, by real-time quantitative PCR (qPCR). Injection with *E. coli* or *Ecc15* induced the expression of all antimicrobial peptide genes tested (Figures 1F–1J) except for the antifungal peptide gene *Drosomycin*, which in turn was only upregulated after infection with *Micrococcus luteus* (*M. luteus*) (Figure 1K).

**Figure 2:**
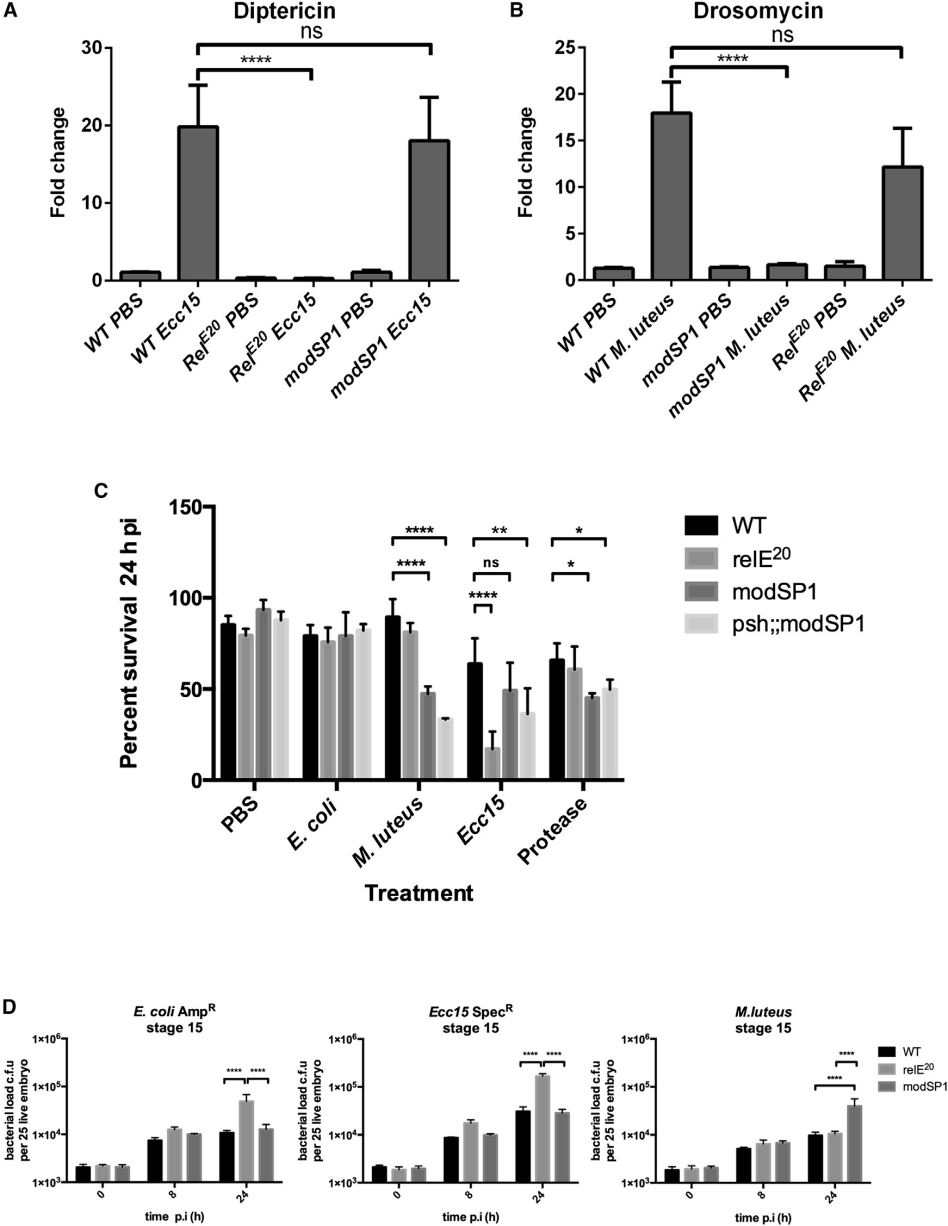
Stage 15 *Drosophila* Embryos Are Able to Effectively Distinguish between Different Types of Infection (A) Real-time qPCR analysis of *Diptericin* expression in stage 15 embryos 2 hr after *Ecc15* infection in the wild-type and *Relish* (*RelA*^*E20*^) and *modular serine protease* (*modSP*^*1*^) mutants shows a clear requirement for Imd signaling in the response to *Ecc15*. (B) Real-time qPCR showing that the expression of *Drosomycin* in stage 15 embryos infected with *M. luteus* depends on the Toll signaling component *modSP*^*1*^. (C) Percentage survival 24 hpi of *RelA*^*E20*^, *modSP*^*1*^, and *psh;modSP*^*1*^ embryos infected with the Gram-positive bacteria *M. luteus*, the Gram-negative bacteria *Ecc15* and *E. coli*, and an *Aspergillus fumigatus* protease cocktail compared with PBS-injected wild-type embryos. ^∗^p < 0.05, ^∗∗^p < 0.01, and ^∗∗∗∗^p < 0.001 as determined by two-way ANOVA followed by an ad hoc Tukey’s multiple comparison test. n = 100 embryos for all genotypes. (D) Bacterial load in infected stage 15 embryos. Bacterial load is controlled in wild-type embryos, but not in *RelA*^*E20*^ or *modSP*^*1*^ embryos. Infections were performed in groups of 25 embryos and reproduced in at least six independent experiments. ^∗∗∗∗^p < 0.001 as determined by determined by two-way ANOVA followed by an ad hoc Tukey’s multiple comparison test.

To ascertain whether the differential response in the embryo was mediated via the Toll and Imd signaling pathways, we assessed *Diptericin* (*Dpt*) and *Drosomycin* (*Drs*) expression in response to *Ecc15* and *M. luteus* injection in embryos mutant for either the Imd signaling component *Relish* (*Rel*^*E20*^) or the Toll signaling component *modular serine protease* (*modSP*^*1*^). *Dpt* expression was significantly diminished in *Rel*^*E20*^ embryos after infection with *Ecc15* in comparison to wild-type levels (Figure 2A), demonstrating a clear requirement for Imd signaling in the immune response to DAP-type PGN stimulation at this early time in the infection. Moreover, this effect on *Dpt* is specific to *Ecc15* infection, as injection with neither the carrier nor *M. luteus* seems to affect the levels of *Dpt* transcript in *Rel*^*E20*^ mutant embryos (Figure 2A). Similarly, *modSP*^*1*^ embryos injected with *M. luteus* fail to upregulate expression of *Drs*, confirming the importance of Toll signaling in mounting an immune response to Lys-type PGN in the embryo (Figure 2B).

The absence of Imd and Toll signaling was also shown to impact the viability of stage 15 embryos after septic injury. The survival of *Rel*^*E20*^, *modSP*^*1*^, and *persephone*^*1*^ (*psh*^*1*^);*modSP*^*1*^ double mutant embryos was monitored 24 hr after injection with different microbial stimuli (Figure 2C). Neither the damage caused by the injection process nor the infection affected the survival of wild-type embryos. All mutant embryos appear to survive infection with *E. coli*; however, injection with *M. luteus* selectively and significantly reduced the survival of *modSP*^*1*^ and *psh*^*1*^;*modSP*^*1*^ double mutant embryos, confirming that the Toll pathway is necessary for the resolution of Gram-positive infections at this stage of *Drosophila* development. *Ecc15* infection decreased the viability not only of *Rel*^*E20*^ but also of *psh*^*1*^;*modSP*^*1*^ double mutant embryos, despite the fact that *modSP*^*1*^ mutant embryos are still able to induce similar levels of diptericin at the early stage of infection and are able to resist infection with *Ecc15* (Figures 2A and 2C), highlighting the contribution of the Toll pathway in resistance to *Ecc15*-induced damage at this stage of development. This result is in accordance with a previous study in which naturally occurring polymorphisms in Toll pathway intracellular signaling components, such as Cactus and Dif, are necessary to contain a systemic infection with the Gram-negative pathogen *Serratia marcescens* [14]. Although this mechanism is still obscure, several studies have proposed a possible crosstalk between the proteolytic cascades that regulate the Toll pathway and those regulating the melanization reaction localizing to the trachea [15–18]. Both *modSP*^*1*^ and *psh*^*1*^;*modSP*^*1*^ double mutant embryos were also less fit compared to wild-type embryos after injection with *Aspergillus oryzae* proteases. We then sought to determine the efficacy of the immune response upon different infections. *Rel*^*E20*^ and *modSP*^*1*^ mutant embryos were infected with *E. coli*, *Ecc15*, and *M. luteus*, and viable bacterial load was measured by quantitative plating at 8 and 24 hr after infection (Figure 2D). Higher bacterial loads were observed in mutant embryos only at 24 hr after infection confirming the importance of Imd and Toll pathways in controlling the infection. Taken together, these results demonstrate that stage 15 embryos are able to suppress infection between 8 and 24 hr after injection and that these responses are mediated via the well-characterized Imd and Toll signaling pathways that have been shown to play a crucial role in the systemic immune response of *Drosophila* larvae and adults.

### Early-Stage Embryos Have a Compromised Immune Response

**Figure 3:**
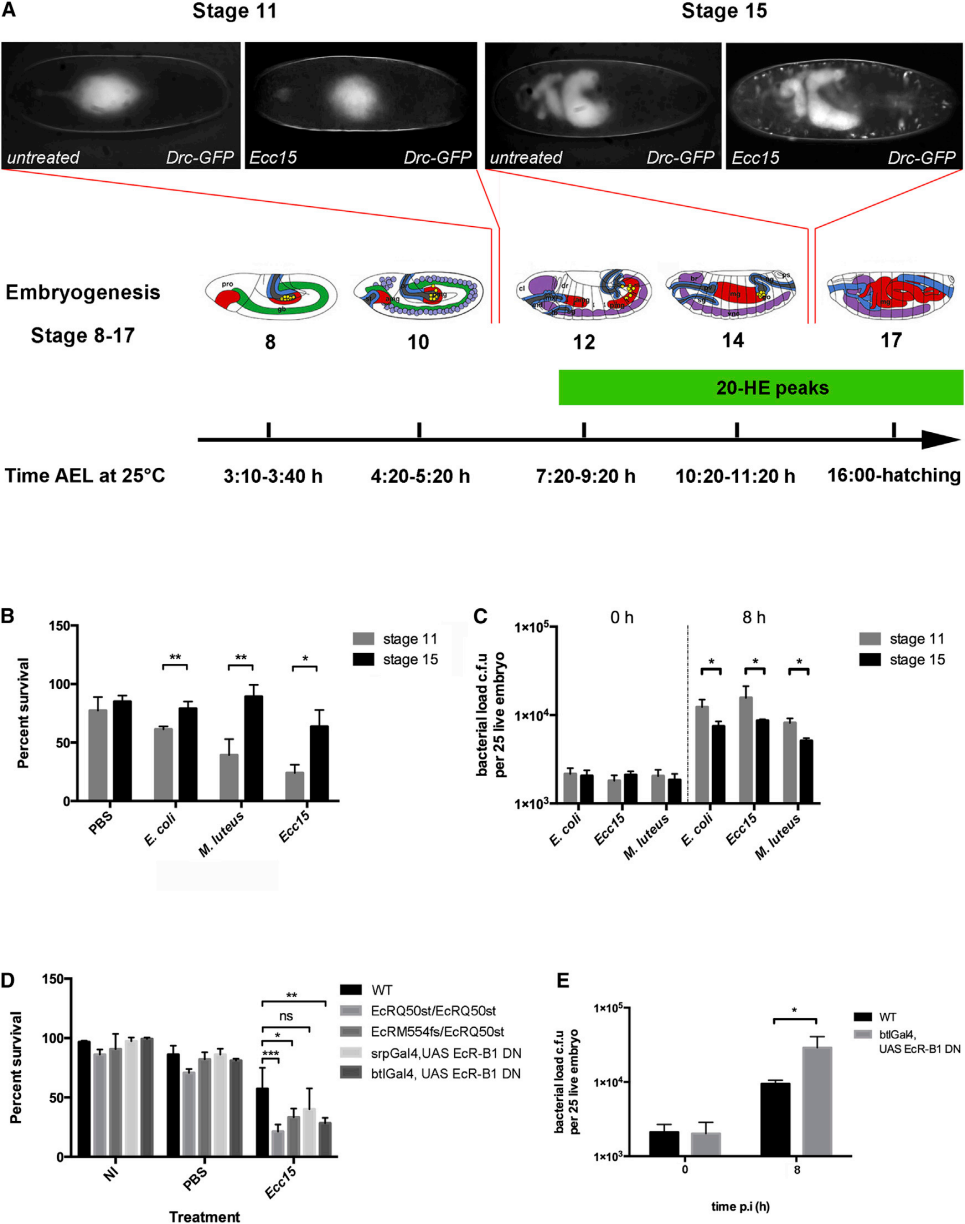
Stage 11 Embryos Show Reduced Immune Competency in Response to Bacterial Invasion (A) Stage 11 embryos expressing Drosocin-GFP fail to switch on Drosocin upon superficial injection with *Ecc15* (compare to stage 15). This development of immune competence coincides with a pulse of ecdysone in the embryo that peaks at approximately 8 hr after egg laying. (B) Survival analysis upon septic injection with *E. coli*, *Ecc15*, and *M. luteus* in stage 15 and stage 11 wild-type embryos clearly shows that early embryos are compromised in their survival after infection with all bacteria tested. Statistical significance was determined by multiple unpaired t tests (^∗^p < 0.05, ^∗∗^p < 0.01). n = 100 embryos for all genotypes. (C) Stage 11 and stage 15 embryos were injected with *Ecc15*, *E. coli*, or *M. luteus*, and colony-forming units were determined at 8 hpi. Bacterial load is significantly higher in infected stage 11 embryos. The significance was assessed by multiple unpaired t tests (p < 0.01). The infections were performed in groups of 25 embryos and reproduced in six independent experiments. (D) Effect of bacterial infection upon survival of stage 15 ecdysone receptor mutant embryos shows that mutants have compromised survival at 24 hpi with *Ecc15*. The survival of embryos expressing dominant-negative EcR-B1 receptor in hemocytes (*srp>EcR-B1 DN*) was not significantly different from that of wild-type embryos, whereas expression of dominant-negative EcR-B1 receptor in the trachea using btl-Gal4 leads to a reduction in survival to levels observed in *EcR* mutants. Statistical significance was determined by two-way ANOVA followed by Tukey’s multiple comparison test (^∗^p < 0.05, ^∗∗^p < 0.01, and ^∗∗∗^p < 0.005). n = 100 embryos for all genotypes. (E) Bacterial load is higher in infected stage 15 *btl>EcR-B1 DN* embryos than in control embryos at 8 hpi. Statistical significance was determined by multiple unpaired t tests (^∗^p = 0.004).

To assess whether embryos at other stages of embryogenesis are able to control infection, we focused our analysis on the AMP responses of embryos at stage 11 of development to Gram-negative infection. In stark contrast to stage 15 embryos, stage 11 embryos fail to express *Drosocin* upon infection with *Ecc15* (Figure 3A). This finding initially seemed at odds with previous studies that have shown yolk-specific Cecropin expression to be activated upon PBS and bacterial injections in early-stage embryos [19]. To investigate this apparent discrepancy in more detail, we sought to determine the potential contribution of the type of injection on early-stage embryos. Using a Cecropin-LacZ fusion line [19], we injected early-stage embryos with endotoxin-free PBS or *Ecc15* either deeply (causing damage to the yolk) or superficially (as all other injections performed in this study). Consistent with previous studies [19], induction of cecropin in the yolk was clearly observed after deep injections, but no expression was seen after superficial injections (Figure S1A available online). Interestingly, the yolk-specific expression of Cecropin was triggered after deep injections whether bacteria were present or not. We also examined AMP gene induction in early-stage embryos after deep injection, confirming the induction of Cecropin under these conditions (Figure S1C). However, we saw no change in the expression of Attacin A, Diptericin, or Drosocin (Figures S1B, S1D, and S1E, respectively) after injection. These results demonstrate that while early embryos are unable to raise an immune response to infection, the yolk appears primed to trigger robust Cecropin expression in response to damage, reminiscent of the damage-induced AMP response previously demonstrated in late-stage embryos [8].

Stage 11 embryos were also not able to contain an infection, as revealed by monitoring the bacterial load as early as 8 hpi (Figure 3C) and their survival to the first-instar larval stage postinfection with bacterial species considered to be nonpathogenic in larvae and adults (Figure 3B). The inability of stage 11 embryos to control infection could reflect either a faster bacterial growth in younger embryos attributable to ample nutrient availability or equally a difference in cellular and humoral resistance mechanisms employed by embryos at different developmental stages.

### Ecdysone Mediates Immune Development in the Embryo

Several studies have suggested that 20-hydroxyecdysone (20-HE) affects the innate immune response of *Drosophila* [20–27]. These studies have shown that 20-HE enhances the expression of AMP genes in infected cultured cell lines and animals. The positive regulatory effects of 20-HE on the Imd pathway have recently been shown to be mediated by at least two mechanisms: one in which 20-HE regulates the expression of the peptidoglycan receptor PGRP-LC, and a second PGRP-LC-independent mechanism that regulates the expression of specific AMPs, including *Dpt*, *Drs*, and *Mtk*, via the transcription factors *Broad complex* (*Br-C*), *Serpent* (*Srp*), and *Pannier* (*Pnr*) [24]. Pulses of 20-HE act as cues for initiating developmental and physiological transitions [28], and one such pulse occurs during embryogenesis after completion of gastrulation and the formation of organ primordia at 6–10 hr of development, with a peak at 8 hr (approximately stage 12 of embryogenesis) [29–31] (Figure 3A). Given the difference in immune competence we observe between stage 11 and stage 15 embryos, we reasoned that the maturation of the immune system might be dependent on this ecdysone pulse. Responses to ecdysone are transduced by a heteromeric nuclear receptor, consisting of the ecdysone receptor (EcR) and the fly ortholog of the vertebrate retinoid X receptor (RXR), Ultraspiracle (Usp) [32, 33]. To test whether ecdysone was mediating the development of immune competence, we analyzed the immune capability of embryos mutant for the EcR receptor.

**Figure 4:**
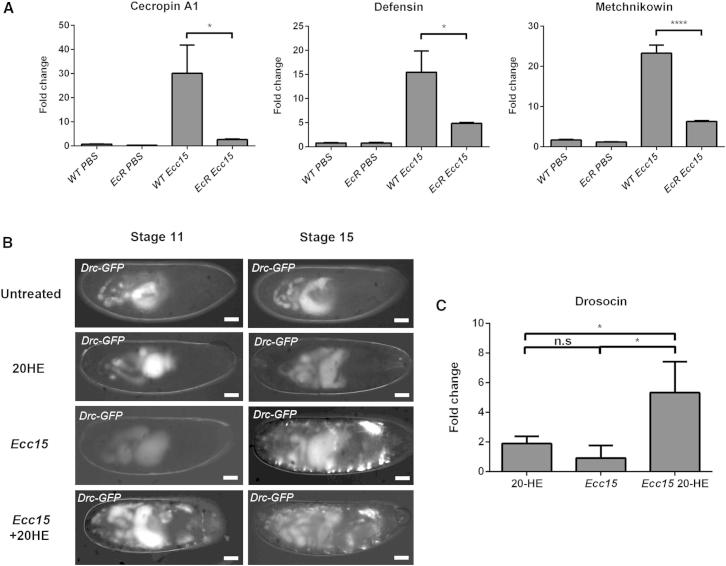
Ecdysone Regulates Embryonic Immune Responses (A) Real-time qPCR analysis of *Cecropin A1*, *Defensin*, and *Metchnikowin* expression in stage 15 wild-type and transheterozygous *EcRQ*^*50st*^*/EcR*^*M55fs*^ mutant embryos at 2 hpi with *Ecc15*. Graphs show a reduced expression of all three AMPs in the mutant after infection. Gene expression levels were normalized to *rp49* levels and were then standardized to nontreated samples and presented as fold change. For each treatment, the values shown represent the mean of three independent experiments. Error bars represent the SD. ^∗^p < 0.05, ^∗∗^p < 0.01, and ^∗∗∗^p < 0.001 as determined by one-way ANOVA with an ad hoc Tukey’s multiple comparison test. (B) Representative images of *Drosocin*-GFP-expressing stage 11 and stage 15 embryos 12 and 6 hpi, respectively, with *Ecc15*. Images show an apparent lack of Drc-GFP expression in young embryos upon bacterial infection (*Ecc15*), which can be rescued upon coinjection with 25 μM ecdysone (*Ecc15+*20HE). Treatment with ecdysone in the absence of bacteria did not cause an upregulation of Drosocin (20-HE). Scale bars represent 50 μm. (C) Real-time qPCR analysis of *Drosocin* expression in stage 11 embryos after treatment with 25 μM 20-HE. The graph shows that, consistent with Drc-GFP data in (B), addition of ecdysone is able to rescue Drosocin expression in early embryos upon *Ecc15* infection. Error bars represent the SD. ^∗^p < 0.05 as determined by one-way ANOVA with an ad hoc Holm-Sidak’s multiple comparison test.

We first verified whether stage 15 embryos containing mutations in EcR were viable. Noninfected stage 15 heterozygous (*EcRQ*^*50st*^*/CTG, EcR*^*M55fs*^*/CTG*), homozygous (*EcRQ*^*50st*^*/EcRQ*^*50st*^*, EcR*^*M55fs*^*/EcR*^*M55fs*^), and transheterozygous (*EcRQ*^*50st*^*/EcR*^*M55fs*^) ecdysone receptor mutant embryos were monitored for development to first-instar larvae. *EcRQ*^*50st*^ mutation affects expression of the EcR-B1 isoform, whereas *EcR*^*M55fs*^ mutation is in a common exon and consequently affects all three EcR isoforms of the EcR protein [34]. Heterozygous, transheterozygous, and homozygous *EcRQ*^*50st*^ mutant embryos did not show a significant difference in viability in comparison to wild-type embryos (Figure 3D). In contrast, *EcR*^*M55fs*^ homozygous mutants were less fit, with only a small percentage of them hatching into first-instar larvae (data not shown). We then tested the susceptibility of stage 15 *EcR*^*Q50st*^ homozygous and *EcRQ*^*50st*^*/EcR*^*M55fs*^ transheterozygous mutant embryos to *Ecc15* infection. Survival of *EcR*^*Q50st*^ homozygous and *EcRQ*^*50st*^*/EcR*^*M55fs*^ transheterozygous mutant embryos was significantly compromised by *Ecc15* infection compared to wild-type survival at 24 hpi (Figure 3D), similar to the viability of embryos injected with *Ecc15* at stage 11 of development (compare with Figure 3B). Furthermore, infection of stage 15 *EcRQ*^*50st*^*/EcR*^*M55fs*^ transheterozygous mutant embryos with *Ecc15* failed to induce the expression of three AMP genes: *Cecropin*, *Defensin*, and *Metchnikowin* (Figure 4A).

The fat body is the *Drosophila* functional equivalent of the mammalian liver and has been implicated as the major immune organ, responding to systemic invasion by secreting AMPs into the hemolymph [12, 35]. Since the fat body only matures at larval stages and given the fact that we do not observe AMP production in the developing fat body in infected embryos, we sought to determine where in the embryo the ecdysone signal is required in controlling immune function. Embryonic hemocytes are the *Drosophila* equivalent of the vertebrate macrophage and have been shown to efficiently clear bacteria at sites of infection in the embryo [9]. Recent studies have shown a requirement for ecdysone signaling within pupal hemocytes for their efficient clearance of some bacteria [36]. To test whether the ecdysone signaling was required in embryonic hemocytes, we expressed a dominant-negative form of EcR-B1 in hemocytes under the control of the hemocytes-specific promoter *srp* and followed the survival of these embryos after *Ecc15* infection. Expression of EcR-B1 in hemocytes compromised the viability of embryos; however, this effect was not statistically significant compared to wild-type embryos (Figure 3D). While expression of EcR-B1 in hemocytes had a minor effect, expression of the same construct in the trachea severely compromised the ability of the embryos to control the infection as early as 8 hpi (Figure 3 E) and contributed to significant killing at later stages such that viability was reduced to the level seen in EcR mutants (Figure 3D). These results show that it is within the tracheal epithelium, the site where AMP production is first observed after infection (Figure 1A), that ecdysone signaling is required and highlights the importance of the tracheal epithelium in the embryonic immune response to infection.

Our results demonstrate a clear requirement for ecdysone signaling in mediating the development of the immune response in embryos, but is ecdysone alone sufficient to confer immune competence? To test this, we treated stage 11 embryos with ecdysone, infected them with *Ecc15*, and monitored their ability to express the AMP *Drosocin*. Remarkably, we found that ecdysone-treated stage 11 embryos were able to upregulate *Drosocin* to similar levels to infected stage 15 embryos (Figures 4B and 4C), demonstrating that edcysone alone is indeed able to confer immune competence in vivo and further highlights the importance of this steroid hormone in mediating the maturation of the immune system in the fly.

In this study, we have addressed the embryonic immune response mechanism in the fly. Our results demonstrate that the embryo uses the Imd and Toll signaling pathways to mediate protective immune responses to bacterial infections. We show that the developing barrier tracheal epithelium is the primary embryonic tissue responding to infection and demonstrate that the ecdysone pulse at stage 12 of embryogenesis is fundamental for the maturation of the immune system of the embryo, with a precocious dose of the hormone being sufficient to confer immune competence to early embryos. Further studies using simple in vivo models such as the *Drosophila* embryo are critical if we are to understand more clearly the role of steroid hormone signaling in mediating inflammation and immunity in vivo.

## Experimental Procedures

### Fly Stocks

*Drosophila* stocks were maintained at 22°C for all experiments. *w;srp-Gal4,UAS-GFP;crq-Gal4,UAS-GFP* were used as wild-type flies. *Rel*^*E20*^*, modSP*^*1*^ *psh*^*1*^;;*modSP*^*1*^, *EcRQ*^*50st*^*/CTG, EcR*^*M55fs*^*/CTG* mutant fly lines and the *Drosocin-GFP* and Cecropin-lacZ lines have been described previously [14, 15, 19, 34, 37–40]. Expression studies with the GAL4/UAS system were carried out using *UAS*-*EcR*-*DN* (*UAS*-*EcR.*^*B1*−*ΔC655.W650A*^) [38].

A detailed description of the methods is included in the Supplemental Experimental Procedures.

## Author Contributions

K.L.T., I.V., and W.W. conceived and designed the experiments. K.L.T. and I.V. performed the experiments. K.L.T., I.V., and W.W. analyzed the data. W.W. and I.V. wrote the manuscript.

## Supplemental Information

Document S1. Figure S1 and Supplemental Experimental Procedures

Document S2. Article plus Supplemental Information
