# Supplementary material for: Ecdysone Mediates the Development of Immunity in the Drosophila Embryo
Source: Curr Biol. 2014 May 19;24(10):1145–52. doi: 10.1016/j.cub.2014.03.062 (PMC4030305; doi:10.1016/j.cub.2014.03.062)
Supplement: Document S1. Figure S1 and Supplemental Experimental Procedures [file mmc1.pdf]

Current Biology, Volume *24*

Supplemental Information

Ecdysone Mediates the Development of Immunity in the *Drosophila* Embryo

Kiri Louise Tan, Isabella Vlisidou, and Will Wood

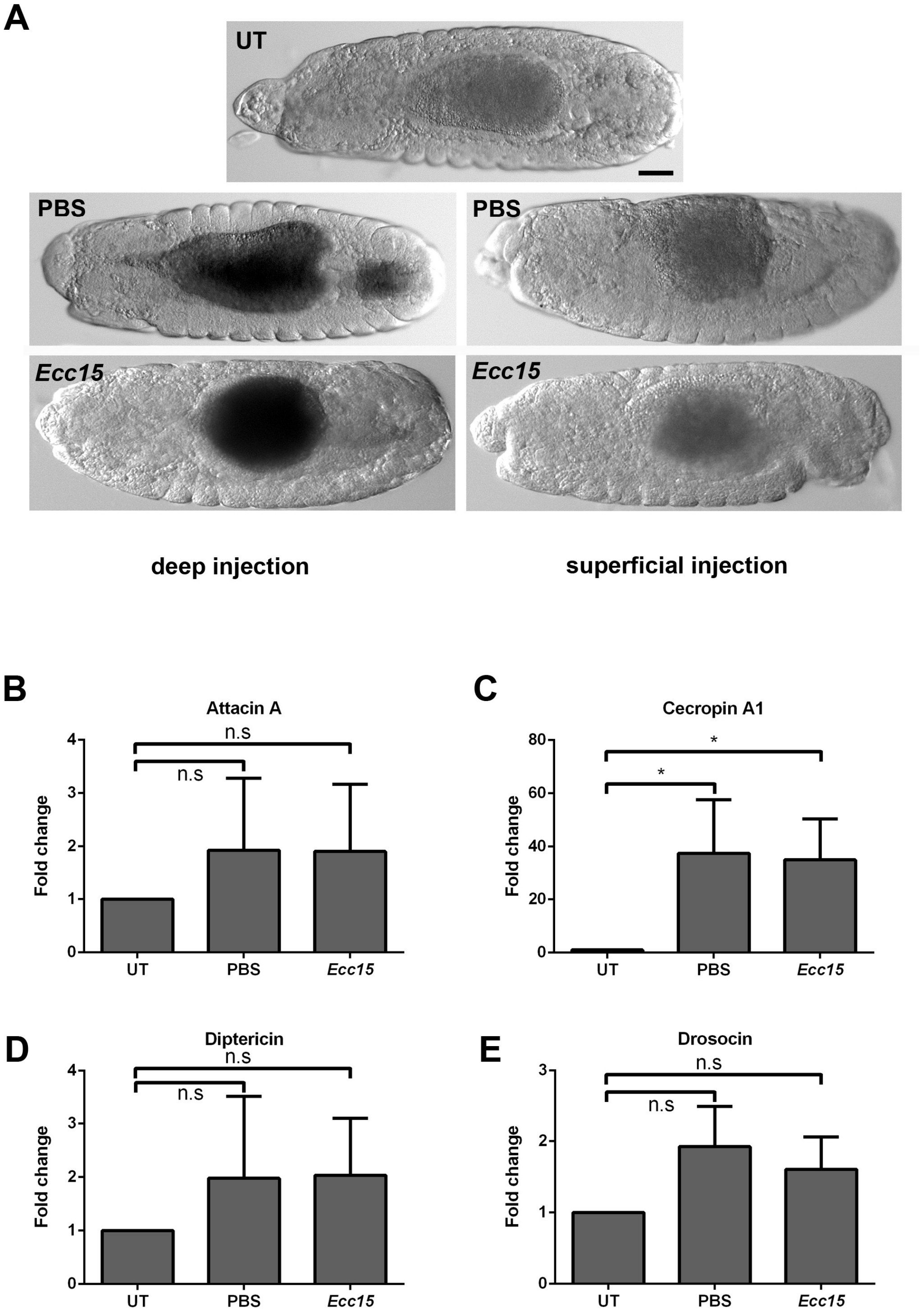

Figure S1. Related to Results and Discussion section; Early stage embryos have a compromised immune response.

(A) Stage 11 embryos carrying a cecropin-LacZ fusion (line described in [S1]) were injected with *Ecc15* in two ways; deep and superficially and incubated at 25°C until they reached stage 12-13. Deep injections lead to activation of the Cecropin in the yolk independently of the inoculum suggesting that physical damage can activate a localised cecropin expression. Scale bars = 50 µm. Attacin A (B), Cecropin A1 (C), Diptericin (D) and Drosocin (E) expression in stage 11 embryos following deep injection of PBS and *Ecc15*. Embryos were treated similarly to (A). Error bars represent St. Dev and *p< 0.05, as determined by one-way ANOVA with an *ad hoc* Holm-Sidak's multiple comparison test.

Supplemental Experimental Procedures.

Bacterial strains and elicitors

All bacteria were grown as shaking cultures in Luria–Bertani medium for 16 h: *Erwinia carotovora carotovora 15* (*Ecc15*) (generously provided by Bruno Lemaitre) at 29°C, *Escherichia coli MG1655* and *Micrococcus luteus* at 37°C. Antibiotic resistant *E. coli* and *Ecc15* were maintained in 100 µg/ml ampicillin and 25 µg/ml spectinomycin respectively. Bacterial strains were used at OD_{600} 1 and endotoxin-free PBS was used as a carrier and for the preparation of the inoculum. In the 20-HE rescue experiment, *Ecc15* infections were performed using an inoculum of OD_{600} 0.5.

Embryo microinjection

Embryos were mounted and subsequently injected using the Eppendorf FemtoJet microinjector platform. An Eppendorf needle was loaded with 5µl of the appropriate culture or control endotoxin-free PBS and the tip broken on the edge of the coverslip

containing the embryos to allow consistent flow of the culture. Embryos subsequently received a superficial injection in the anterior region of approximately 3nl of culture or control PBS for 1 second at an injection pressure (P_i) of 50-100 hPa and compensation pressure (P_c) of 20hPa. This injection typically delivers 100-150 bacteria per embryo. Post injection, embryos were incubated at 22°C in a box containing moistened tissue for the appropriate time period. 20-HE treatment was performed by injection of the hormone from a stock of 25 μM. Several studies have attempted treatments of whole embryos, embryonic-derived cells and larval or pupae tissues with concentrations ranging from 1 μM -5μM [S2-S6]. As injection of ecdysone in *Drosophila* embryos has not been described before and taking into account the variability of the injection process, we tested a variety of 20-HE concentrations for defects in germ band retraction and consistent AMP up-regulation. A concentration as high as 25 μM did not affect germ band retraction and embryonic development and it reproducibly upregulates AMP expression (to a minimum of approximately 30%).

Determination of bacterial load

Infected embryos were released from the microinjection slide by a brief wash with heptane and followed by washes with sterile PBS. Bacterial load was measured by homogenising 25 live embryos or larvae in 200 μl sterile PBS and quantitatively plating 25 μl homogenate on LB agar plates with appropriate antibiotics. Plates were grown at appropriate temperatures until colonies were visible. Homogenates from embryos injected with sterile PBS yield no colonies. In total, the infections were repeated in at least 6 independent experiments for each genotype and bacterial species

combination. Larvae were scored as dead if they did not respond to gentle prodding with a Tungsten needle and if the dorsal vessel did not beat.

Quantitative RT-PCR

For quantification of *AMP* mRNA, whole embryos were collected at 2 h post injection (p.i) by a brief heptane wash. Total embryo RNA was isolated from 200 embryos using the QIAzol reagent and the RNEasy column kit and dissolved in 50 µl of RNase-free water. DNase treatment of RNA samples was undertaken to remove any genomic DNA present using Turbo DNAse kit (Ambion). The RNA was then transferred to a clean tube and the concentration subsequently determined using the Qubit Quantification Platform and Quant-iT assay kit (Invitrogen). One microgram of total RNA was then reverse-transcribed in 25 µl reaction volume using the Superscript III First-Strand Synthesis System for Real-Time quantitative-PCR (Invitrogen) and oligo-d$(T)_{20}$ primers. Quantitative PCR was performed on a Step One Plus qPCR system (ABI) in 96-well plates using the SYBR Green I master mix (BIORAD). Primers sequences are as follows: *Cecropin A1* forward 5'-GAACTTCTACAACATCTTCGT-3' and reverse 5'-TCCCAGTCCCTGGATT-3'; *Defensin* forward 5'-GTTCTTCGTTCTCGTGG-3' and reverse 5'-CTTTGAACCCCTTGGC-3'; *Diptericin* forward 5'-GCTGCGCAATCGCTTCTACT-3' and reverse 5'-TGGTGGAGTGGGCTTCATG-3'; *Drosocin* forward 5'-CCATCGTTTTCCTGCT-3' and reverse 5'-CTTGAGTCAGGTGATCC-3'; *Drosomycin* forward 5'-CGTGAGAACCTTTTCCAATATGATG-3' and reverse 5'-TCCCAGGACCACCAGCAT-3'; *Metchnikowin* forward 5'-AACTTAATCTTGGAGCGA-3' and reverse 5'-CGGTCTTGGTTGGTTAG-3';

Rp49 forward 5'-GACGCTTCAAGGGACAGTATCTG-3' and reverse 5'-AAACGCGGTTCTGCATGAG-3'.

Immunohistochemistry, live imaging and confocal microscopy

Stage 15 embryos were dechorionated and fixed as previously described [S7]. The embryonic vitelline membrane was removed by hand. Excess PBS was removed and the embryos were dehydrated in methanol in PBS. Embryos were then permeabilised in 0.1% Triton-X in PBS (PBT) before being washed in 0.1% Triton X, 1% BSA in PBS (PATx) to block non-specific staining. Embryos were incubated overnight at 4°C in primary antibodies diluted to the required concentration in PATx, (anti-GFP in 1:500, anti-trachea 2A12 in 1:20) before further washes with PATx. Samples were then incubated with secondary antibodies at required concentrations for 2 hours at room temperature, further washed with PATx and transferred to 1,4-diazabiycylo[2.2.2]octane (DABCO) for imaging. Live detection of GFP expression and localisation on whole embryos was performed using a Leica M716F fluorescence dissecting scope, Leica DC350FX camera and Adobe Photoshop C53 software linked to a TWAIN module for Leica DC cameras. For visualisation of *Drc-GFP* expression on antibody stained embryos a Perkin Elmer spinning disk microscope was employed using Volocity Image Analysis software (v.6.3).

Statistical analysis

All analyses were performed using GraphPad Prism software version 6. Data were subjected to appropriate ANOVA with ad hoc Tukey's or Holm-Sidak's multiple comparison tests.

Supplemental References.

S1. Tingvall, T.O., Roos, E., and Engstrom, Y. (2001). The GATA factor Serpent is required for the onset of the humoral immune response in Drosophila embryos. Proc Natl Acad Sci U S A *98*, 3884-3888.

S2. Andres, A.J., and Cherbas, P. (1992). Tissue-specific ecdysone responses: regulation of the Drosophila genes Eip28/29 and Eip40 during larval development. Development *116*, 865-876.

S3. Kozlova, T., and Thummel, C.S. (2002). Spatial patterns of ecdysteroid receptor activation during the onset of Drosophila metamorphosis. Development *129*, 1739-1750.

S4. Kozlova, T., and Thummel, C.S. (2003). Essential roles for ecdysone signaling during Drosophila mid-embryonic development. Science *301*, 1911-1914.

S5. Savakis, C., Koehler, M.M., and Cherbas, P. (1984). cDNA clones for the ecdysone-inducible polypeptide (EIP) mRNAs of Drosophila Kc cells. EMBO J *3*, 235-243.

S6. Tsurumi, A., Dutta, P., Yan, S.J., Sheng, R., and Li, W.X. (2013). Drosophila Kdm4 demethylases in histone H3 lysine 9 demethylation and ecdysteroid signaling. Sci Rep *3*, 2894.

S7. Evans, I.R., Hu, N., Skaer, H., and Wood, W. (2010). Interdependence of macrophage migration and ventral nerve cord development in Drosophila embryos. Development *137*, 1625-1633.
