# Supplementary material for: Ecdysone Mediates the Development of Immunity in the Drosophila Embryo
Source: Curr Biol. 2014 May 19;24(10):1145–52. doi: 10.1016/j.cub.2014.03.062 (PMC4030305; doi:10.1016/j.cub.2014.03.062)
Supplement: Document S2. Article plus Supplemental Information [file mmc2.pdf]

Current Biology 24, 1145–1152, May 19, 2014 ©2014 The Authors http://dx.doi.org/10.1016/j.cub.2014.03.062

Report

Ecdysone Mediates the Development of Immunity in the *Drosophila* Embryo

Kiri Louise Tan,[1,2] Isabella Vlisidou,[1,2] and Will Wood[1,*]
[1]Faculty of Medical and Veterinary Sciences, University of Bristol, University Walk, Bristol BS8 1TD, UK

Summary

Beyond their role in cell metabolism, development, and reproduction, hormones are also important modulators of the immune system. In the context of inflammatory disorders, systemic administration of pharmacological doses of synthetic glucocorticoids (GCs) is widely used as an anti-inflammatory treatment [1, 2]. However, not all actions of GCs are immunosuppressive, and many studies have suggested that physiological concentrations of GCs can have immunoenhancing effects [3–7]. For a more comprehensive understanding of how steroid hormones regulate immunity and inflammation, a simple in vivo system is required. The *Drosophila* embryo has recently emerged as a powerful model system to study the recruitment of immune cells to sterile wounds [8] and host-pathogen dynamics [9]. Here we investigate the immune response of the fly embryo to bacterial infections and find that the steroid hormone 20-hydroxyecdysone (20-HE) can regulate the quality of the immune response and influence the resolution of infection in *Drosophila* embryos.

Results and Discussion

Drosophila Embryos Can Mount an Immune Response to Bacterial Challenge

Using a previously established embryo microinjection assay [9], we first sought to determine whether late-stage *Drosophila* embryos are able to induce a humoral immune response after septic injury. The humoral response to microbe infection in *Drosophila* is largely mediated by two pathways: Imd and Toll. Pathogen recognition is initiated by pattern recognition receptors that bind conserved stereotypical, rather than particular, molecular structures present in a wide spectrum of microorganisms but absent in the host [10], such as peptidoglycan (PGN), which is a major constituent of the cell wall of both Gram-negative and Gram-positive bacteria [11]. *Drosophila* senses mesodiaminopimelic acid (DAP)-type PGN present in a single layer within the Gram-negative bacteria periplasmic space via two peptidoglycan recognition proteins (PGRPs), the membrane-bound PGRP-LC and the secreted and/or cytosolic PGRP-LE, activating the Imd signaling pathway. Sensing of the Lys-type peptidoglycan present on the surface of Gram-positive bacteria is mediated by PGRP-SA, PGRP-SD, and Gram-negative binding protein 1 (GNBP1), which relay the signal to the Toll pathway [12]. A subclass of Gram-positive bacteria including *Bacillus* species and *Listeria monocytogenes* also produce DAP-type PGN, which renders them able to activate the Imd signaling pathway [12]. Activation of antimicrobial peptide (AMP) genes and quantification of pathogen load are widely used in both larval and adult fly models as readouts of the immune response. As a proxy for AMP production, we monitored the expression of a *Drosocin-gfp* (*Drc*-GFP) promoter fusion construct in stage 15 embryos injected with either *Escherichia coli* (*E. coli*) or *Erwinia carotovora carotovora 15* (*Ecc15*) [13].

Infection with *E. coli* or *Ecc15* initially induces *Drc*-GFP transgene expression throughout the tracheal system as early as 3 hr postinfection (hpi) (Figure 1A). This is followed by a later expression in the epithelium at 6 hpi (Figures 1D and 1E). The microinjection process itself had no effect on Drosocin expression (Figure 1C). To assess the early effects of septic injury on stage 15 embryos in more detail, we analyzed the transcriptional response of several AMP genes, including *Cecropin A1*, *Defensin*, *Diptericin*, *Drosocin*, *Drosomycin*, and *Metchnikowin*, by real-time quantitative PCR (qPCR). Injection with *E. coli* or *Ecc15* induced the expression of all antimicrobial peptide genes tested (Figures 1F–1J) except for the antifungal peptide gene *Drosomycin*, which in turn was only upregulated after infection with *Micrococcus luteus* (*M. luteus*) (Figure 1K).

To ascertain whether the differential response in the embryo was mediated via the Toll and Imd signaling pathways, we assessed *Diptericin* (*Dpt*) and *Drosomycin* (*Drs*) expression in response to *Ecc15* and *M. luteus* injection in embryos mutant for either the Imd signaling component *Relish* (Rel^{E20}) or the Toll signaling component *modular serine protease* ($modSP^1$). *Dpt* expression was significantly diminished in Rel^{E20} embryos after infection with *Ecc15* in comparison to wild-type levels (Figure 2A), demonstrating a clear requirement for Imd signaling in the immune response to DAP-type PGN stimulation at this early time in the infection. Moreover, this effect on *Dpt* is specific to *Ecc15* infection, as injection with neither the carrier nor *M. luteus* seems to affect the levels of *Dpt* transcript in Rel^{E20} mutant embryos (Figure 2A). Similarly, $modSP^1$ embryos injected with *M. luteus* fail to upregulate expression of *Drs*, confirming the importance of Toll signaling in mounting an immune response to Lys-type PGN in the embryo (Figure 2B).

The absence of Imd and Toll signaling was also shown to impact the viability of stage 15 embryos after septic injury. The survival of Rel^{E20}, $modSP^1$, and *persephone*^{1} (psh^1); $modSP^1$ double mutant embryos was monitored 24 hr after injection with different microbial stimuli (Figure 2C). Neither the damage caused by the injection process nor the infection affected the survival of wild-type embryos. All mutant embryos appear to survive infection with *E. coli*; however, injection with *M. luteus* selectively and significantly reduced the survival of $modSP^1$ and psh^1;$modSP^1$ double mutant embryos, confirming that the Toll pathway is necessary for the resolution of Gram-positive infections at this stage of *Drosophila* development. *Ecc15* infection decreased the viability not only of Rel^{E20} but also of psh^1;$modSP^1$ double mutant embryos, despite the fact that $modSP^1$ mutant embryos are still able to induce similar levels of diptericin at the early stage of infection and are able to resist infection with *Ecc15* (Figures 2A and 2C), highlighting the contribution of the Toll pathway in resistance to *Ecc15*-induced damage at this stage of development.

[2]Co-first author
*Correspondence: w.wood@bristol.ac.uk
This is an open access article under the CC BY license (http://creativecommons.org/licenses/by/3.0/).

CrossMark

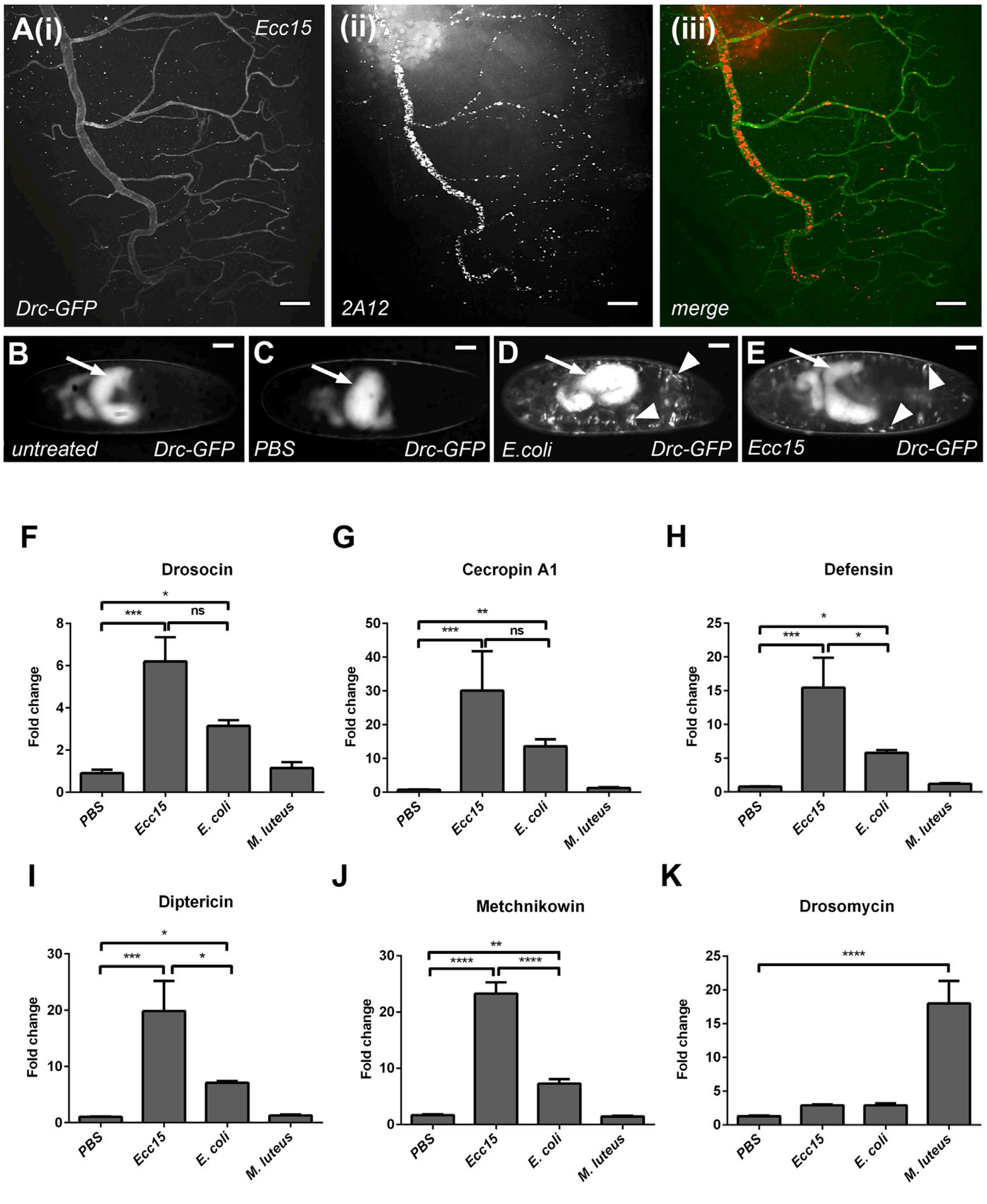

Figure 1. Stage 15 *Drosophila* Embryos Are Able to Mount Immune Responses to Bacterial Challenge

(A–E) A stage 15 embryo expressing Drosocin-GFP stained with anti-GFP (Ai) and the tracheal-specific antibody 2A12 (Aii). The merge (Aiii) shows clear Drc expression throughout the tracheal network 3 hpi with *Ecc15*. A live, untreated, Drc-GFP-expressing embryo (B) and a Drc-GFP-expressing embryo (C) 6 hpi with PBS show no Drc expression, whereas injection with either *E. coli* (D) or *Ecc15* (E) leads to robust expression in the embryonic epithelium (arrowheads). Arrows show autofluorescence in the yolk. Scale bars represent 20 μm (A) and 50 μm (B–E).

(F–K) Real-time qPCR analysis of *Drosocin* (F), *Cecropin A1* (G), *Defensin* (H), *Diptericin* (I), *Metchnikowin* (J), and *Drosomycin* (K) in stage 15 embryos injected with endotoxin-free PBS or live bacterial cells of *E. coli*, *M. luteus*, and *E. carotovora* (*Ecc15*) for 2 hr. The expression of antimicrobial peptide genes was normalized to the reference gene *rp49* and then standardized to the expression level of nontreated samples. The mean of three independent biological replicates is shown, and error bars represent the SD. *$p < 0.05$, **$p < 0.01$, and p*** < 0.001 as determined by one-way ANOVA with an ad hoc Tukey's multiple comparison test. n = 200 embryos.

This result is in accordance with a previous study in which naturally occurring polymorphisms in Toll pathway intracellular signaling components, such as Cactus and Dif, are necessary to contain a systemic infection with the Gram-negative pathogen *Serratia marcescens* [14]. Although this mechanism is still obscure, several studies have proposed a possible crosstalk between the proteolytic cascades that regulate the Toll pathway and those regulating the

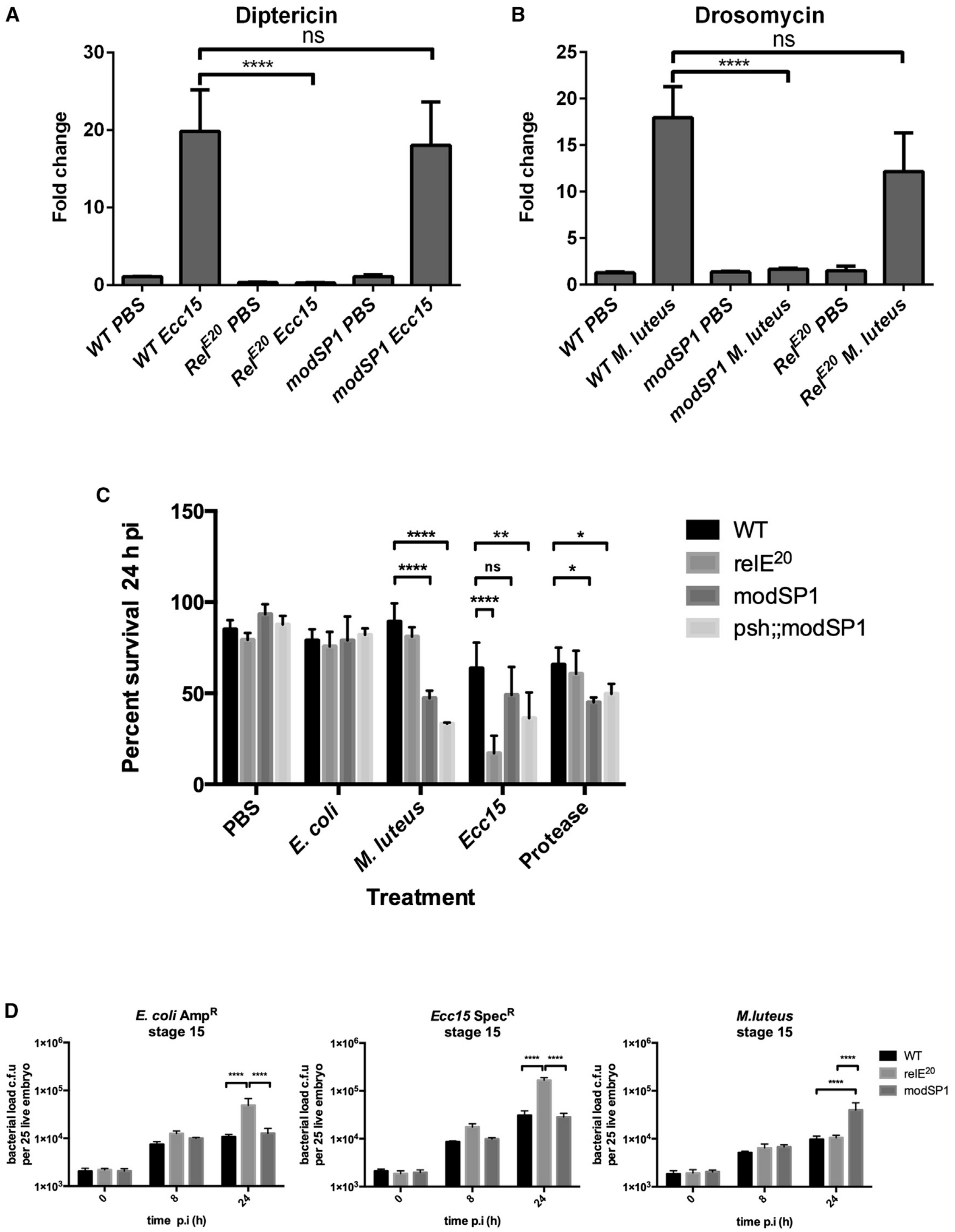

Figure 2. Stage 15 *Drosophila* Embryos Are Able to Effectively Distinguish between Different Types of Infection

(A) Real-time qPCR analysis of *Diptericin* expression in stage 15 embryos 2 hr after *Ecc15* infection in the wild-type and *Relish* ($RelA^{E20}$) and *modular serine protease* ($modSP^{1}$) mutants shows a clear requirement for Imd signaling in the response to *Ecc15*.

(B) Real-time qPCR showing that the expression of *Drosomycin* in stage 15 embryos infected with *M. luteus* depends on the Toll signaling component $modSP^{1}$.

(C) Percentage survival 24 hpi of $RelA^{E20}$, $modSP^{1}$, and $psh;modSP^{1}$ embryos infected with the Gram-positive bacteria *M. luteus*, the Gram-negative bacteria *Ecc15* and *E. coli*, and an *Aspergillus fumigatus* protease cocktail compared with PBS-injected wild-type embryos. *$p < 0.05$, **$p < 0.01$, and ****$p < 0.001$ as determined by two-way ANOVA followed by an ad hoc Tukey's multiple comparison test. n = 100 embryos for all genotypes.

(D) Bacterial load in infected stage 15 embryos. Bacterial load is controlled in wild-type embryos, but not in $RelA^{E20}$ or $modSP^{1}$ embryos. Infections were performed in groups of 25 embryos and reproduced in at least six independent experiments. ****$p < 0.001$ as determined by determined by two-way ANOVA followed by an ad hoc Tukey's multiple comparison test.

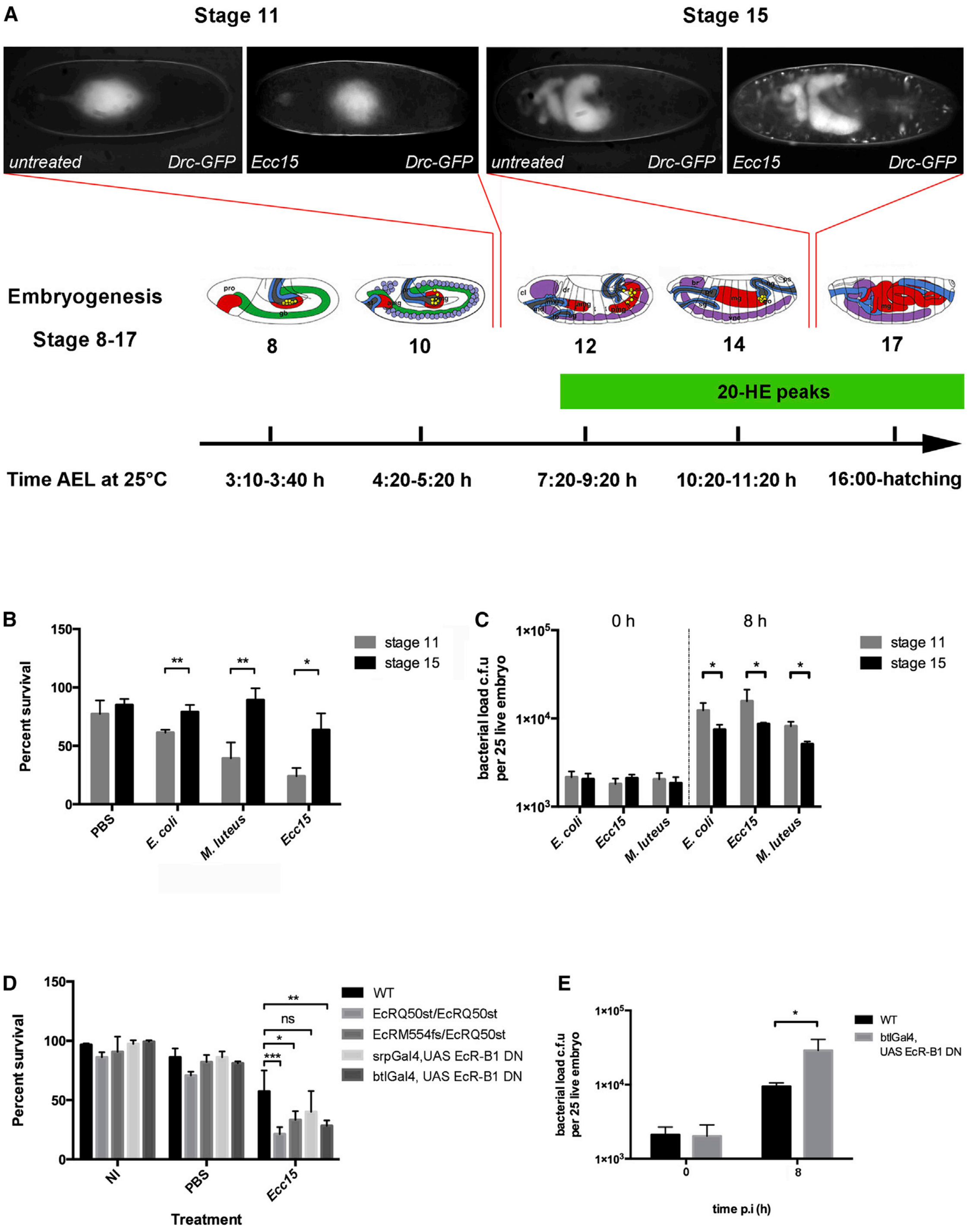

Figure 3. Stage 11 Embryos Show Reduced Immune Competency in Response to Bacterial Invasion

(A) Stage 11 embryos expressing Drosocin-GFP fail to switch on Drosocin upon superficial injection with *Ecc15* (compare to stage 15). This development of immune competence coincides with a pulse of ecdysone in the embryo that peaks at approximately 8 hr after egg laying.

(B) Survival analysis upon septic injection with *E. coli*, *Ecc15*, and *M. luteus* in stage 15 and stage 11 wild-type embryos clearly shows that early embryos are compromised in their survival after infection with all bacteria tested. Statistical significance was determined by multiple unpaired t tests (*$p < 0.05$, **$p < 0.01$). n = 100 embryos for all genotypes.

(C) Stage 11 and stage 15 embryos were injected with *Ecc15*, *E. coli*, or *M. luteus*, and colony-forming units were determined at 8 hpi. Bacterial load is significantly higher in infected stage 11 embryos. The significance was assessed by multiple unpaired t tests ($p < 0.01$). The infections were performed in groups of 25 embryos and reproduced in six independent experiments.

(D) Effect of bacterial infection upon survival of stage 15 ecdysone receptor mutant embryos shows that mutants have compromised survival at 24 hpi with *Ecc15*. The survival of embryos expressing dominant-negative EcR-B1 receptor in hemocytes (*srp>EcR-B1 DN*) was not significantly different from that of wild-type embryos, whereas expression of dominant-negative EcR-B1 receptor in the trachea using btl-Gal4 leads to a reduction in survival to levels

(legend continued on next page)

melanization reaction localizing to the trachea [15–18]. Both *modSP*^{1} and *psh*^{1};*modSP*^{1} double mutant embryos were also less fit compared to wild-type embryos after injection with *Aspergillus oryzae* proteases. We then sought to determine the efficacy of the immune response upon different infections. *Rel*^{E20} and *modSP*^{1} mutant embryos were infected with *E. coli*, *Ecc15*, and *M. luteus*, and viable bacterial load was measured by quantitative plating at 8 and 24 hr after infection (Figure 2D). Higher bacterial loads were observed in mutant embryos only at 24 hr after infection confirming the importance of Imd and Toll pathways in controlling the infection. Taken together, these results demonstrate that stage 15 embryos are able to suppress infection between 8 and 24 hr after injection and that these responses are mediated via the well-characterized Imd and Toll signaling pathways that have been shown to play a crucial role in the systemic immune response of *Drosophila* larvae and adults.

Early-Stage Embryos Have a Compromised Immune Response

To assess whether embryos at other stages of embryogenesis are able to control infection, we focused our analysis on the AMP responses of embryos at stage 11 of development to Gram-negative infection. In stark contrast to stage 15 embryos, stage 11 embryos fail to express *Drosocin* upon infection with *Ecc15* (Figure 3A). This finding initially seemed at odds with previous studies that have shown yolk-specific Cecropin expression to be activated upon PBS and bacterial injections in early-stage embryos [19]. To investigate this apparent discrepancy in more detail, we sought to determine the potential contribution of the type of injection on early-stage embryos. Using a Cecropin-LacZ fusion line [19], we injected early-stage embryos with endotoxin-free PBS or *Ecc15* either deeply (causing damage to the yolk) or superficially (as all other injections performed in this study). Consistent with previous studies [19], induction of cecropin in the yolk was clearly observed after deep injections, but no expression was seen after superficial injections (Figure S1A available online). Interestingly, the yolk-specific expression of Cecropin was triggered after deep injections whether bacteria were present or not. We also examined AMP gene induction in early-stage embryos after deep injection, confirming the induction of Cecropin under these conditions (Figure S1C). However, we saw no change in the expression of Attacin A, Diptericin, or Drosocin (Figures S1B, S1D, and S1E, respectively) after injection. These results demonstrate that while early embryos are unable to raise an immune response to infection, the yolk appears primed to trigger robust Cecropin expression in response to damage, reminiscent of the damage-induced AMP response previously demonstrated in late-stage embryos [8].

Stage 11 embryos were also not able to contain an infection, as revealed by monitoring the bacterial load as early as 8 hpi (Figure 3C) and their survival to the first-instar larval stage postinfection with bacterial species considered to be nonpathogenic in larvae and adults (Figure 3B). The inability of stage 11 embryos to control infection could reflect either a faster bacterial growth in younger embryos attributable to ample nutrient availability or equally a difference in cellular and humoral resistance mechanisms employed by embryos at different developmental stages.

Ecdysone Mediates Immune Development in the Embryo

Several studies have suggested that 20-hydroxyecdysone (20-HE) affects the innate immune response of *Drosophila* [20–27]. These studies have shown that 20-HE enhances the expression of AMP genes in infected cultured cell lines and animals. The positive regulatory effects of 20-HE on the Imd pathway have recently been shown to be mediated by at least two mechanisms: one in which 20-HE regulates the expression of the peptidoglycan receptor PGRP-LC, and a second PGRP-LC-independent mechanism that regulates the expression of specific AMPs, including *Dpt*, *Drs*, and *Mtk*, via the transcription factors *Broad complex* (*Br-C*), *Serpent* (*Srp*), and *Pannier* (*Pnr*) [24]. Pulses of 20-HE act as cues for initiating developmental and physiological transitions [28], and one such pulse occurs during embryogenesis after completion of gastrulation and the formation of organ primordia at 6–10 hr of development, with a peak at 8 hr (approximately stage 12 of embryogenesis) [29–31] (Figure 3A). Given the difference in immune competence we observe between stage 11 and stage 15 embryos, we reasoned that the maturation of the immune system might be dependent on this ecdysone pulse. Responses to ecdysone are transduced by a heteromeric nuclear receptor, consisting of the ecdysone receptor (EcR) and the fly ortholog of the vertebrate retinoid X receptor (RXR), Ultraspiracle (Usp) [32, 33]. To test whether ecdysone was mediating the development of immune competence, we analyzed the immune capability of embryos mutant for the EcR receptor.

We first verified whether stage 15 embryos containing mutations in EcR were viable. Noninfected stage 15 heterozygous (*EcRQ*^{50st}/*CTG*, *EcR*^{M55fs}/*CTG*), homozygous (*EcRQ*^{50st}/*EcRQ*^{50st}, *EcR*^{M55fs}/*EcR*^{M55fs}), and transheterozygous (*EcRQ*^{50st}/*EcR*^{M55fs}) ecdysone receptor mutant embryos were monitored for development to first-instar larvae. *EcRQ*^{50st} mutation affects expression of the EcR-B1 isoform, whereas *EcR*^{M55fs} mutation is in a common exon and consequently affects all three EcR isoforms of the EcR protein [34]. Heterozygous, transheterozygous, and homozygous *EcRQ*^{50st} mutant embryos did not show a significant difference in viability in comparison to wild-type embryos (Figure 3D). In contrast, *EcR*^{M55fs} homozygous mutants were less fit, with only a small percentage of them hatching into first-instar larvae (data not shown). We then tested the susceptibility of stage 15 *EcR*^{Q50st} homozygous and *EcRQ*^{50st}/*EcR*^{M55fs} transheterozygous mutant embryos to *Ecc15* infection. Survival of *EcR*^{Q50st} homozygous and *EcRQ*^{50st}/*EcR*^{M55fs} transheterozygous mutant embryos was significantly compromised by *Ecc15* infection compared to wild-type survival at 24 hpi (Figure 3D), similar to the viability of embryos injected with *Ecc15* at stage 11 of development (compare with Figure 3B). Furthermore, infection of stage 15 *EcRQ*^{50st}/*EcR*^{M55fs} transheterozygous mutant embryos with *Ecc15* failed to induce the expression of three AMP genes: *Cecropin*, *Defensin*, and *Metchnikowin* (Figure 4A).

The fat body is the *Drosophila* functional equivalent of the mammalian liver and has been implicated as the major immune

observed in *EcR* mutants. Statistical significance was determined by two-way ANOVA followed by Tukey's multiple comparison test (*p < 0.05, **p < 0.01, and ***p < 0.005). n = 100 embryos for all genotypes.

(E) Bacterial load is higher in infected stage 15 *btl>EcR-B1 DN* embryos than in control embryos at 8 hpi. Statistical significance was determined by multiple unpaired t tests (*p = 0.004).

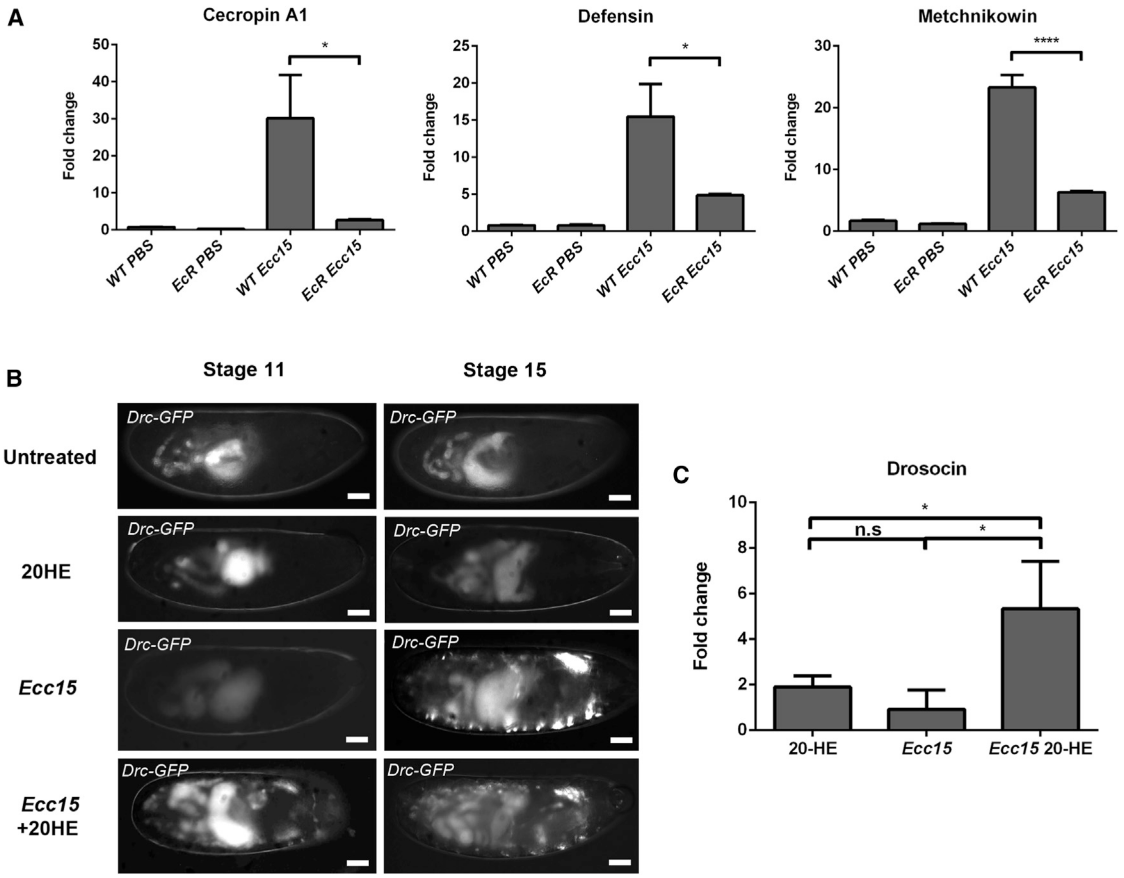

Figure 4. Ecdysone Regulates Embryonic Immune Responses

(A) Real-time qPCR analysis of *Cecropin A1*, *Defensin*, and *Metchnikowin* expression in stage 15 wild-type and transheterozygous $EcRQ^{50st}/EcR^{M55fs}$ mutant embryos at 2 hpi with *Ecc15*. Graphs show a reduced expression of all three AMPs in the mutant after infection. Gene expression levels were normalized to *rp49* levels and were then standardized to nontreated samples and presented as fold change. For each treatment, the values shown represent the mean of three independent experiments. Error bars represent the SD. *p < 0.05, **p < 0.01, and ***p < 0.001 as determined by one-way ANOVA with an ad hoc Tukey's multiple comparison test.

(B) Representative images of *Drosocin*-GFP-expressing stage 11 and stage 15 embryos 12 and 6 hpi, respectively, with *Ecc15*. Images show an apparent lack of Drc-GFP expression in young embryos upon bacterial infection (*Ecc15*), which can be rescued upon coinjection with 25 μM ecdysone (*Ecc15*+20HE). Treatment with ecdysone in the absence of bacteria did not cause an upregulation of Drosocin (20-HE). Scale bars represent 50 μm.

(C) Real-time qPCR analysis of *Drosocin* expression in stage 11 embryos after treatment with 25 μM 20-HE. The graph shows that, consistent with Drc-GFP data in (B), addition of ecdysone is able to rescue Drosocin expression in early embryos upon *Ecc15* infection. Error bars represent the SD. *p < 0.05 as determined by one-way ANOVA with an ad hoc Holm-Sidak's multiple comparison test.

organ, responding to systemic invasion by secreting AMPs into the hemolymph [12, 35]. Since the fat body only matures at larval stages and given the fact that we do not observe AMP production in the developing fat body in infected embryos, we sought to determine where in the embryo the ecdysone signal is required in controlling immune function. Embryonic hemocytes are the *Drosophila* equivalent of the vertebrate macrophage and have been shown to efficiently clear bacteria at sites of infection in the embryo [9]. Recent studies have shown a requirement for ecdysone signaling within pupal hemocytes for their efficient clearance of some bacteria [36]. To test whether the ecdysone signaling was required in embryonic hemocytes, we expressed a dominant-negative form of EcR-B1 in hemocytes under the control of the hemocytes-specific promoter *srp* and followed the survival of these embryos after *Ecc15* infection. Expression of EcR-B1 in hemocytes compromised the viability of embryos; however, this effect was not statistically significant compared to wild-type embryos (Figure 3D). While expression of EcR-B1 in hemocytes had a minor effect, expression of the same construct in the trachea severely compromised the ability of the embryos to control the infection as early as 8 hpi (Figure 3 E) and contributed to significant killing at later stages such that viability was reduced to the level seen in EcR mutants (Figure 3D). These results show that it is within the tracheal epithelium, the site where AMP production is first observed after infection (Figure 1A), that ecdysone signaling is required and highlights the importance of the tracheal epithelium in the embryonic immune response to infection.

Our results demonstrate a clear requirement for ecdysone signaling in mediating the development of the immune response in embryos, but is ecdysone alone sufficient to confer immune competence? To test this, we treated stage 11 embryos with ecdysone, infected them with *Ecc15*, and monitored their ability to express the AMP *Drosocin*. Remarkably, we found that ecdysone-treated stage 11 embryos were able to upregulate *Drosocin* to similar levels to infected stage 15 embryos (Figures 4B and 4C), demonstrating that edcysone

alone is indeed able to confer immune competence in vivo and further highlights the importance of this steroid hormone in mediating the maturation of the immune system in the fly.

In this study, we have addressed the embryonic immune response mechanism in the fly. Our results demonstrate that the embryo uses the Imd and Toll signaling pathways to mediate protective immune responses to bacterial infections. We show that the developing barrier tracheal epithelium is the primary embryonic tissue responding to infection and demonstrate that the ecdysone pulse at stage 12 of embryogenesis is fundamental for the maturation of the immune system of the embryo, with a precocious dose of the hormone being sufficient to confer immune competence to early embryos. Further studies using simple in vivo models such as the *Drosophila* embryo are critical if we are to understand more clearly the role of steroid hormone signaling in mediating inflammation and immunity in vivo.

Experimental Procedures

Fly Stocks

Drosophila stocks were maintained at 22°C for all experiments. *w;srp-Gal4,UAS-GFP;crq-Gal4,UAS-GFP* were used as wild-type flies. Rel^{E20}, $modSP^{1}$ psh^{1};;$modSP^{1}$, $EcRQ^{50st}$/CTG, EcR^{M55fs}/CTG mutant fly lines and the *Drosocin-GFP* and Cecropin-lacZ lines have been described previously [14, 15, 19, 34, 37–40]. Expression studies with the GAL4/UAS system were carried out using *UAS-EcR-DN* ($UAS\text{-}EcR.^{B1-\Delta C655.W650A}$) [38].

A detailed description of the methods is included in the Supplemental Experimental Procedures.

Supplemental Information

Supplemental Information includes one figure and Supplemental Experimental Procedures and can be found with this article online at http://dx.doi.org/10.1016/j.cub.2014.03.062.

Author Contributions

K.L.T., I.V., and W.W. conceived and designed the experiments. K.L.T. and I.V. performed the experiments. K.L.T., I.V., and W.W. analyzed the data. W.W. and I.V. wrote the manuscript.

Acknowledgments

We would like to thank Bruno Lemaitre and the Bloomington Drosophila Stock Center for fly lines and the Developmental Studies Hybridoma Bank for antibodies. W.W. is funded by a Senior Research Fellowship from the Wellcome Trust.

Received: October 11, 2013
Revised: February 6, 2014
Accepted: March 24, 2014
Published: May 1, 2014

References

1. McColl, A., Michlewska, S., Dransfield, I., and Rossi, A.G. (2007). Effects of glucocorticoids on apoptosis and clearance of apoptotic cells. ScientificWorldJournal *7*, 1165–1181.
2. Necela, B.M., and Cidlowski, J.A. (2004). Mechanisms of glucocorticoid receptor action in noninflammatory and inflammatory cells. Proc. Am. Thorac. Soc. *1*, 239–246.
3. Busillo, J.M., Azzam, K.M., and Cidlowski, J.A. (2011). Glucocorticoids sensitize the innate immune system through regulation of the NLRP3 inflammasome. J. Biol. Chem. *286*, 38703–38713.
4. Galon, J., Franchimont, D., Hiroi, N., Frey, G., Boettner, A., Ehrhart-Bornstein, M., O'Shea, J.J., Chrousos, G.P., and Bornstein, S.R. (2002). Gene profiling reveals unknown enhancing and suppressive actions of glucocorticoids on immune cells. FASEB J. *16*, 61–71.
5. Hermoso, M.A., Matsuguchi, T., Smoak, K., and Cidlowski, J.A. (2004). Glucocorticoids and tumor necrosis factor alpha cooperatively regulate toll-like receptor 2 gene expression. Mol. Cell. Biol. *24*, 4743–4756.
6. Sakai, A., Han, J., Cato, A.C., Akira, S., and Li, J.D. (2004). Glucocorticoids synergize with IL-1beta to induce TLR2 expression via MAP Kinase Phosphatase-1-dependent dual Inhibition of MAPK JNK and p38 in epithelial cells. BMC Mol. Biol. *5*, 2.
7. Shuto, T., Imasato, A., Jono, H., Sakai, A., Xu, H., Watanabe, T., Rixter, D.D., Kai, H., Andalibi, A., Linthicum, F., et al. (2002). Glucocorticoids synergistically enhance nontypeable Haemophilus influenzae-induced Toll-like receptor 2 expression via a negative cross-talk with p38 MAP kinase. J. Biol. Chem. *277*, 17263–17270.
8. Stramer, B., Winfield, M., Shaw, T., Millard, T.H., Woolner, S., and Martin, P. (2008). Gene induction following wounding of wild-type versus macrophage-deficient Drosophila embryos. EMBO Rep. *9*, 465–471.
9. Vlisidou, I., Dowling, A.J., Evans, I.R., Waterfield, N., ffrench-Constant, R.H., and Wood, W. (2009). Drosophila embryos as model systems for monitoring bacterial infection in real time. PLoS Pathog. *5*, e1000518.
10. Medzhitov, R., and Janeway, C.A., Jr. (2002). Decoding the patterns of self and nonself by the innate immune system. Science *296*, 298–300.
11. Leulier, F., Parquet, C., Pili-Floury, S., Ryu, J.H., Caroff, M., Lee, W.J., Mengin-Lecreulx, D., and Lemaitre, B. (2003). The Drosophila immune system detects bacteria through specific peptidoglycan recognition. Nat. Immunol. *4*, 478–484.
12. Lemaitre, B., and Hoffmann, J. (2007). The host defense of Drosophila melanogaster. Annu. Rev. Immunol. *25*, 697–743.
13. Basset, A., Khush, R.S., Braun, A., Gardan, L., Boccard, F., Hoffmann, J.A., and Lemaitre, B. (2000). The phytopathogenic bacteria Erwinia carotovora infects Drosophila and activates an immune response. Proc. Natl. Acad. Sci. USA *97*, 3376–3381.
14. Lazzaro, B.P., Sceurman, B.K., and Clark, A.G. (2004). Genetic basis of natural variation in D. melanogaster antibacterial immunity. Science *303*, 1873–1876.
15. Buchon, N., Poidevin, M., Kwon, H.M., Guillou, A., Sottas, V., Lee, B.L., and Lemaitre, B. (2009). A single modular serine protease integrates signals from pattern-recognition receptors upstream of the Drosophila Toll pathway. Proc. Natl. Acad. Sci. USA *106*, 12442–12447.
16. Ligoxygakis, P., Pelte, N., Hoffmann, J.A., and Reichhart, J.M. (2002). Activation of Drosophila Toll during fungal infection by a blood serine protease. Science *297*, 114–116.
17. Ligoxygakis, P., Pelte, N., Ji, C., Leclerc, V., Duvic, B., Belvin, M., Jiang, H., Hoffmann, J.A., and Reichhart, J.M. (2002). A serpin mutant links Toll activation to melanization in the host defence of Drosophila. EMBO J. *21*, 6330–6337.
18. Tang, H., Kambris, Z., Lemaitre, B., and Hashimoto, C. (2008). A serpin that regulates immune melanization in the respiratory system of Drosophila. Dev. Cell *15*, 617–626.
19. Tingvall, T.O., Roos, E., and Engström, Y. (2001). The GATA factor Serpent is required for the onset of the humoral immune response in Drosophila embryos. Proc. Natl. Acad. Sci. USA *98*, 3884–3888.
20. Dimarcq, J.L., Imler, J.L., Lanot, R., Ezekowitz, R.A., Hoffmann, J.A., Janeway, C.A., and Lagueux, M. (1997). Treatment of l(2)mbn Drosophila tumorous blood cells with the steroid hormone ecdysone amplifies the inducibility of antimicrobial peptide gene expression. Insect Biochem. Mol. Biol. *27*, 877–886.
21. Flatt, T., Heyland, A., Rus, F., Porpiglia, E., Sherlock, C., Yamamoto, R., Garbuzov, A., Palli, S.R., Tatar, M., and Silverman, N. (2008). Hormonal regulation of the humoral innate immune response in Drosophila melanogaster. J. Exp. Biol. *211*, 2712–2724.
22. Lanot, R., Zachary, D., Holder, F., and Meister, M. (2001). Postembryonic hematopoiesis in Drosophila. Dev. Biol. *230*, 243–257.
23. Meister, M., and Richards, G. (1996). Ecdysone and insect immunity: the maturation of the inducibility of the diptericin gene in Drosophila larvae. Insect Biochem. Mol. Biol. *26*, 155–160.
24. Rus, F., Flatt, T., Tong, M., Aggarwal, K., Okuda, K., Kleino, A., Yates, E., Tatar, M., and Silverman, N. (2013). Ecdysone triggered PGRP-LC expression controls Drosophila innate immunity. EMBO J. *32*, 1626–1638.
25. Sorrentino, R.P., Carton, Y., and Govind, S. (2002). Cellular immune response to parasite infection in the Drosophila lymph gland is developmentally regulated. Dev. Biol. *243*, 65–80.

26. Stofanko, M., Kwon, S.Y., and Badenhorst, P. (2008). A misexpression screen to identify regulators of Drosophila larval hemocyte development. Genetics *180*, 253–267.
27. Zhang, Z., and Palli, S.R. (2009). Identification of a cis-regulatory element required for 20-hydroxyecdysone enhancement of antimicrobial peptide gene expression in Drosophila melanogaster. Insect Mol. Biol. *18*, 595–605.
28. Kozlova, T., and Thummel, C.S. (2000). Steroid regulation of postembryonic development and reproduction in Drosophila. Trends Endocrinol. Metab. *11*, 276–280.
29. Kozlova, T., and Thummel, C.S. (2003). Essential roles for ecdysone signaling during Drosophila mid-embryonic development. Science *301*, 1911–1914.
30. Kraminsky, G.P., Clark, W.C., Estelle, M.A., Gietz, R.D., Sage, B.A., O'Connor, J.D., and Hodgetts, R.B. (1980). Induction of translatable mRNA for dopa decarboxylase in Drosophila: an early response to ecdysterone. Proc. Natl. Acad. Sci. USA *77*, 4175–4179.
31. Sullivan, A.A., and Thummel, C.S. (2003). Temporal profiles of nuclear receptor gene expression reveal coordinate transcriptional responses during Drosophila development. Mol. Endocrinol. *17*, 2125–2137.
32. Riddiford, L.M., Cherbas, P., and Truman, J.W. (2000). Ecdysone receptors and their biological actions. Vitam. Horm. *60*, 1–73.
33. Thummel, C.S. (1996). Flies on steroids—Drosophila metamorphosis and the mechanisms of steroid hormone action. Trends Genet. *12*, 306–310.
34. Bender, M., Imam, F.B., Talbot, W.S., Ganetzky, B., and Hogness, D.S. (1997). Drosophila ecdysone receptor mutations reveal functional differences among receptor isoforms. Cell *91*, 777–788.
35. Silverman, N., and Maniatis, T. (2001). NF-kappaB signaling pathways in mammalian and insect innate immunity. Genes Dev. *15*, 2321–2342.
36. Regan, J.C., Brandão, A.S., Leitão, A.B., Mantas Dias, A.R., Sucena, E., Jacinto, A., and Zaidman-Rémy, A. (2013). Steroid hormone signaling is essential to regulate innate immune cells and fight bacterial infection in Drosophila. PLoS Pathog. *9*, e1003720.
37. Brückner, K., Kockel, L., Duchek, P., Luque, C.M., Rørth, P., and Perrimon, N. (2004). The PDGF/VEGF receptor controls blood cell survival in Drosophila. Dev. Cell *7*, 73–84.
38. Cherbas, L., Hu, X., Zhimulev, I., Belyaeva, E., and Cherbas, P. (2003). EcR isoforms in Drosophila: testing tissue-specific requirements by targeted blockade and rescue. Development *130*, 271–284.
39. Hedengren, M., Asling, B., Dushay, M.S., Ando, I., Ekengren, S., Wihlborg, M., and Hultmark, D. (1999). Relish, a central factor in the control of humoral but not cellular immunity in Drosophila. Mol. Cell *4*, 827–837.
40. Tzou, P., Ohresser, S., Ferrandon, D., Capovilla, M., Reichhart, J.M., Lemaitre, B., Hoffmann, J.A., and Imler, J.L. (2000). Tissue-specific inducible expression of antimicrobial peptide genes in Drosophila surface epithelia. Immunity *13*, 737–748.

Current Biology, Volume *24*

Supplemental Information

Ecdysone Mediates the Development of Immunity in the *Drosophila* Embryo

Kiri Louise Tan, Isabella Vlisidou, and Will Wood

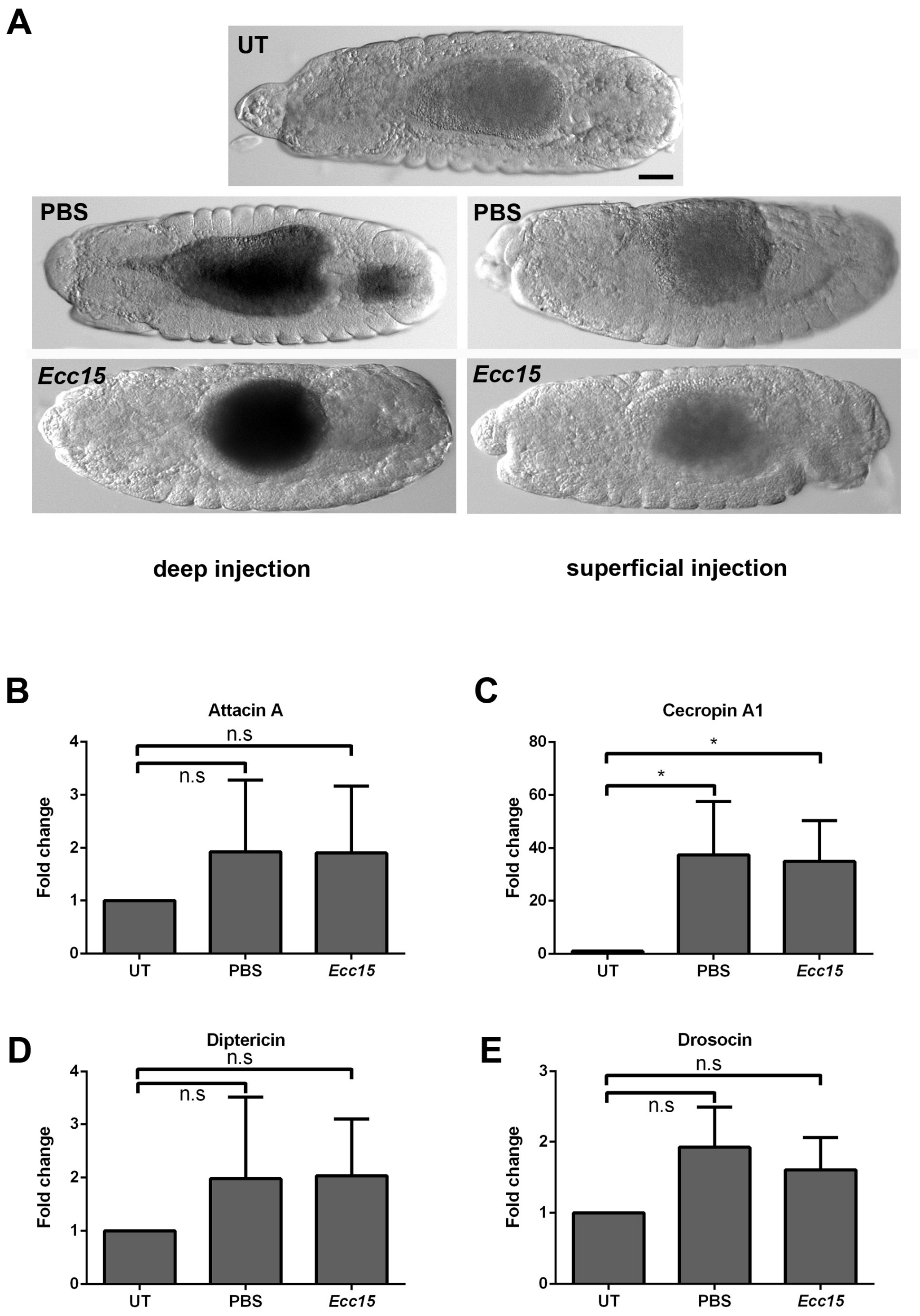

Figure S1. Related to Results and Discussion section; Early stage embryos have a compromised immune response.

(A) Stage 11 embryos carrying a cecropin-LacZ fusion (line described in [S1]) were injected with *Ecc15* in two ways; deep and superficially and incubated at 25°C until they reached stage 12-13. Deep injections lead to activation of the Cecropin in the yolk independently of the inoculum suggesting that physical damage can activate a localised cecropin expression. Scale bars = 50 µm. Attacin A (B), Cecropin A1 (C), Diptericin (D) and Drosocin (E) expression in stage 11 embryos following deep injection of PBS and *Ecc15*. Embryos were treated similarly to (A). Error bars represent St. Dev and *$p < 0.05$, as determined by one-way ANOVA with an *ad hoc* Holm-Sidak's multiple comparison test.

Supplemental Experimental Procedures.

Bacterial strains and elicitors

All bacteria were grown as shaking cultures in Luria–Bertani medium for 16 h: *Erwinia carotovora carotovora 15* (*Ecc15*) (generously provided by Bruno Lemaitre) at 29°C, *Escherichia coli MG1655* and *Micrococcus luteus* at 37°C. Antibiotic resistant *E. coli* and *Ecc15* were maintained in 100 µg/ml ampicillin and 25 µg/ml spectinomycin respectively. Bacterial strains were used at OD_{600} 1 and endotoxin-free PBS was used as a carrier and for the preparation of the inoculum. In the 20-HE rescue experiment, *Ecc15* infections were performed using an inoculum of OD_{600} 0.5.

Embryo microinjection

Embryos were mounted and subsequently injected using the Eppendorf FemtoJet microinjector platform. An Eppendorf needle was loaded with 5µl of the appropriate culture or control endotoxin-free PBS and the tip broken on the edge of the coverslip

containing the embryos to allow consistent flow of the culture. Embryos subsequently received a superficial injection in the anterior region of approximately 3nl of culture or control PBS for 1 second at an injection pressure (P_i) of 50-100 hPa and compensation pressure (P_c) of 20hPa. This injection typically delivers 100-150 bacteria per embryo. Post injection, embryos were incubated at 22°C in a box containing moistened tissue for the appropriate time period. 20-HE treatment was performed by injection of the hormone from a stock of 25 μM. Several studies have attempted treatments of whole embryos, embryonic-derived cells and larval or pupae tissues with concentrations ranging from 1 μM -5μM [S2-S6]. As injection of ecdysone in *Drosophila* embryos has not been described before and taking into account the variability of the injection process, we tested a variety of 20-HE concentrations for defects in germ band retraction and consistent AMP up-regulation. A concentration as high as 25 μM did not affect germ band retraction and embryonic development and it reproducibly upregulates AMP expression (to a minimum of approximately 30%).

Determination of bacterial load

Infected embryos were released from the microinjection slide by a brief wash with heptane and followed by washes with sterile PBS. Bacterial load was measured by homogenising 25 live embryos or larvae in 200 μl sterile PBS and quantitatively plating 25 μl homogenate on LB agar plates with appropriate antibiotics. Plates were grown at appropriate temperatures until colonies were visible. Homogenates from embryos injected with sterile PBS yield no colonies. In total, the infections were repeated in at least 6 independent experiments for each genotype and bacterial species

combination. Larvae were scored as dead if they did not respond to gentle prodding with a Tungsten needle and if the dorsal vessel did not beat.

Quantitative RT-PCR

For quantification of *AMP* mRNA, whole embryos were collected at 2 h post injection (p.i) by a brief heptane wash. Total embryo RNA was isolated from 200 embryos using the QIAzol reagent and the RNEasy column kit and dissolved in 50 µl of RNase-free water. DNase treatment of RNA samples was undertaken to remove any genomic DNA present using Turbo DNAse kit (Ambion). The RNA was then transferred to a clean tube and the concentration subsequently determined using the Qubit Quantification Platform and Quant-iT assay kit (Invitrogen). One microgram of total RNA was then reverse-transcribed in 25 µl reaction volume using the Superscript III First-Strand Synthesis System for Real-Time quantitative-PCR (Invitrogen) and oligo-d(T)$_{20}$ primers. Quantitative PCR was performed on a Step One Plus qPCR system (ABI) in 96-well plates using the SYBR Green I master mix (BIORAD). Primers sequences are as follows: *Cecropin A1* forward 5'-GAACTTCTACAACATCTTCGT-3' and reverse 5'-TCCCAGTCCCTGGATT-3'; *Defensin* forward 5'-GTTCTTCGTTCTCGTGG-3' and reverse 5'-CTTTGAACCCCTTGGC-3'; *Diptericin* forward 5'-GCTGCGCAATCGCTTCTACT-3' and reverse 5'-TGGTGGAGTGGGCTTCATG-3'; *Drosocin* forward 5'-CCATCGTTTTCCTGCT-3' and reverse 5'-CTTGAGTCAGGTGATCC-3'; *Drosomycin* forward 5'-CGTGAGAACCTTTTCCAATATGATG-3' and reverse 5'-TCCCAGGACCACCAGCAT-3'; *Metchnikowin* forward 5'-AACTTAATCTTGGAGCGA-3' and reverse 5'-CGGTCTTGGTTGGTTAG-3';

Rp49 forward 5'-GACGCTTCAAGGGACAGTATCTG-3' and reverse 5'-AAACGCGGTTCTGCATGAG-3'.

Immunohistochemistry, live imaging and confocal microscopy

Stage 15 embryos were dechorionated and fixed as previously described [S7]. The embryonic vitelline membrane was removed by hand. Excess PBS was removed and the embryos were dehydrated in methanol in PBS. Embryos were then permeabilised in 0.1% Triton-X in PBS (PBT) before being washed in 0.1% Triton X, 1% BSA in PBS (PATx) to block non-specific staining. Embryos were incubated overnight at 4°C in primary antibodies diluted to the required concentration in PATx, (anti-GFP in 1:500, anti-trachea 2A12 in 1:20) before further washes with PATx. Samples were then incubated with secondary antibodies at required concentrations for 2 hours at room temperature, further washed with PATx and transferred to 1,4-diazabiycylo[2.2.2]octane (DABCO) for imaging. Live detection of GFP expression and localisation on whole embryos was performed using a Leica M716F fluorescence dissecting scope, Leica DC350FX camera and Adobe Photoshop C53 software linked to a TWAIN module for Leica DC cameras. For visualisation of *Drc-GFP* expression on antibody stained embryos a Perkin Elmer spinning disk microscope was employed using Volocity Image Analysis software (v.6.3).

Statistical analysis

All analyses were performed using GraphPad Prism software version 6. Data were subjected to appropriate ANOVA with ad hoc Tukey's or Holm-Sidak's multiple comparison tests.

Supplemental References.

S1. Tingvall, T.O., Roos, E., and Engstrom, Y. (2001). The GATA factor Serpent is required for the onset of the humoral immune response in Drosophila embryos. Proc Natl Acad Sci U S A *98*, 3884-3888.

S2. Andres, A.J., and Cherbas, P. (1992). Tissue-specific ecdysone responses: regulation of the Drosophila genes Eip28/29 and Eip40 during larval development. Development *116*, 865-876.

S3. Kozlova, T., and Thummel, C.S. (2002). Spatial patterns of ecdysteroid receptor activation during the onset of Drosophila metamorphosis. Development *129*, 1739-1750.

S4. Kozlova, T., and Thummel, C.S. (2003). Essential roles for ecdysone signaling during Drosophila mid-embryonic development. Science *301*, 1911-1914.

S5. Savakis, C., Koehler, M.M., and Cherbas, P. (1984). cDNA clones for the ecdysone-inducible polypeptide (EIP) mRNAs of Drosophila Kc cells. EMBO J *3*, 235-243.

S6. Tsurumi, A., Dutta, P., Yan, S.J., Sheng, R., and Li, W.X. (2013). Drosophila Kdm4 demethylases in histone H3 lysine 9 demethylation and ecdysteroid signaling. Sci Rep *3*, 2894.

S7. Evans, I.R., Hu, N., Skaer, H., and Wood, W. (2010). Interdependence of macrophage migration and ventral nerve cord development in Drosophila embryos. Development *137*, 1625-1633.
